# Exercise-induced modulation of gut microbiota in individuals with obesity and type 2 diabetes: a systematic review and meta-analysis

**DOI:** 10.3389/fmicb.2025.1671975

**Published:** 2025-09-24

**Authors:** Weian Lin, Lei Pu, Xingyu Qian, Jinchen Pan, Ruiqi Cheng, Peng Sun

**Affiliations:** ^1^The Key Laboratory of Adolescent Health Assessment and Exercise Intervention of the Ministry of Education, East China Normal University, Shanghai, China; ^2^China Basketball College, Beijing Sport University, Beijing, China; ^3^School of Athletic Performance, Shanghai University of Sport, Shanghai, China; ^4^College of Physical Education and Health, East China Normal University, Shanghai, China

**Keywords:** diabetes, obesity, gut microbiota, exercise, meta-analysis

## Abstract

**Objective:**

This systematic review and meta-analysis aimed to evaluate the effects of exercise on gut microbiota in individuals with obesity and type 2 diabetes (T2D), including alpha-diversity indices (Shannon, Simpson, Chao1, and observed OTUs) and taxonomic composition, to explore the potential role of gut microbiota in mediating the effects of exercise on disease progression.

**Methods:**

A total of 19 studies comprising 1,062 participants were included. Alpha-diversity indices and taxonomic changes were analyzed using meta-analysis and qualitative synthesis. Subgroup analyses were conducted based on exercise type and age.

**Results:**

Meta-analysis showed that exercise significantly increased the Shannon index in both the obesity group (SMD = 0.40 [0.15, 0.65], *P* = 0.002) and the T2D group (SMD = 0.48 [0.08, 0.88], *P* = 0.02). No significant changes were observed in the Simpson index or observed OTUs. The Chao1 index showed a significant improvement in individuals with obesity (SMD = 0.45 [0.06, 0.85], *P* = 0.03). Subgroup analyses indicated that combined exercise produced more pronounced effects than aerobic exercise alone in both the obesity group (SMD = 0.42, *P* = 0.02) and the T2D group (SMD = 0.69, *P* = 0.04). Younger individuals (<50 years) were more responsive to exercise interventions (Obesity: SMD = 0.32, *P* = 0.027; T2D: SMD = 0.86, *P* = 0.003). Qualitative synthesis revealed consistent enrichment of butyrate-producing taxa (notably Roseburia and *Faecalibacterium prausnitzii*) and *Akkermansia muciniphila*, while responses of the Firmicutes/Bacteroidetes ratio and genus-level taxa such as *Prevotella* and *Bacteroides* varied across studies.

**Conclusion:**

Exercise significantly enhances gut microbiota diversity in individuals with obesity and T2D, with combined exercise showing potentially greater benefits. Younger populations may respond more effectively to exercise interventions. Future research should further investigate the effects of personalized exercise strategies.

**Systematic review registration:**

https://www.crd.york.ac.uk/PROSPERO/view/CRD420251015520, identifier CRD420250653594

## 1 Introduction

Since the 1990s, the prevalence of obesity and its associated metabolic disorders, such as type 2 diabetes (T2D), has risen dramatically. At present, hundreds of millions of individuals worldwide are affected by obesity and T2D, and this number continues to grow (World Health Organization [WHO], 2024a,2024b). Obesity is a major risk factor for T2D, with approximately 80% of patients classified as either obese or overweight (BMI ≥ 25 kg/m^2^) (Jin et al., 2023). Numerous studies have demonstrated a strong positive correlation between obesity and the incidence of T2D, with individuals having a BMI > 30 kg/m^2^ being three to seven times more likely to develop the disease compared to those with normal weight (González-Muniesa et al., 2017; Piché et al., 2020). Moreover, obesity and T2D frequently coexist, constituting a pathological state characterized by mutually reinforcing metabolic abnormalities. Extensive evidence indicates that individuals with obesity and T2D exhibit more profound metabolic impairments, including heightened chronic inflammation, dysregulated lipid metabolism, and exacerbated insulin resistance (Donath et al., 2019; Béliard et al., 2024). This comorbid condition not only accelerates the progression of diabetes but also markedly increases the risk of multiple chronic complications, such as cardiovascular disease and certain types of cancer (American Diabetes Association Professional Practice Committee, 2024), thereby further aggravating the burden on global public health and healthcare systems.

The gut microbiota, comprising trillions of microorganisms residing primarily in the gastrointestinal tract, plays a fundamental role in host metabolism and immune regulation (Patterson et al., 2016; Gomes et al., 2018). Mounting evidence suggests that gut dysbiosis, characterized by reduced microbial diversity and structural imbalance, is a hallmark of both obesity and T2D (Vallianou et al., 2019; Patra et al., 2023). Obesity-related dysbiosis is typically marked by an increased Firmicutes-to-Bacteroidetes ratio and a reduction in microbial gene richness, both of which contribute to metabolic dysfunction, enhanced fat storage, and elevated inflammatory cytokines (Geng et al., 2022; Islam et al., 2023). These microbial alterations compromise gut barrier integrity, facilitating the translocation of harmful metabolic by-products such as lipopolysaccharides (LPS) into the bloodstream. This process triggers systemic inflammation, exacerbates insulin resistance, and ultimately increases the risk of developing T2D (Wu et al., 2020; Liu et al., 2021). Furthermore, individuals with obesity and T2D exhibit significant gut microbiota differences compared to their healthy counterparts, including decreased microbial diversity and an increased abundance of pathogenic bacteria (Pitocco et al., 2020; Yang et al., 2021; Zhang et al., 2021). These dysbiotic alterations contribute to disruptions in bile acid metabolism, a reduction in short-chain fatty acid (SCFA) production, and the exacerbation of endotoxemia, all of which further promote metabolic dysfunction (Lee et al., 2020). Given the pivotal role of the gut microbiota in nutrient absorption, energy homeostasis, and systemic inflammation, modulating the gut microbiota has emerged as a promising therapeutic strategy for managing obesity and T2D.

Exercise is widely recognized as an effective non-pharmacological intervention for the prevention and management of chronic diseases (Chen et al., 2023). Recent studies have highlighted its critical role in shaping gut microbiota composition (Allen et al., 2018), which is particularly relevant to metabolic regulation. Exercise has been shown to enhance gut microbiota diversity, increase the abundance of beneficial bacterial genera, and reduce the proportion of pathogenic bacteria (Liu et al., 2020; Aragón-Vela et al., 2021). These microbial adaptations are considered key mechanisms through which exercise confers systemic health benefits. However, evidence regarding these effects in individuals with metabolic disorders remains limited. Unlike healthy individuals, patients with chronic conditions such as obesity and T2D often exhibit gut microbiota dysbiosis, which may influence their responsiveness to exercise interventions (Pinart et al., 2022). Findings from randomized controlled trials (RCTs) have been inconsistent, with some studies reporting significant improvements in gut microbiota following exercise (Kern et al., 2020; Wei et al., 2022; Cullen et al., 2024; Wang et al., 2024), while others have found no substantial effects (Dupuit et al., 2022; Cullen et al., 2023; Batitucci et al., 2024). This inconsistency underscores the need for further research to elucidate the potential benefits of exercise in restoring gut microbiota homeostasis in individuals with chronic diseases. In addition, the pathophysiological mechanisms in individuals with obesity and T2D may differ from those observed in obesity or T2D alone, particularly with respect to the more intricate interactions involved in maintaining gut microbiota homeostasis. Consequently, focusing on this population and systematically evaluating the modulatory effects of exercise on their gut microbiota holds greater practical significance for intervention and enhanced potential for clinical translation. Therefore, this study conducted a systematic review and meta-analysis to evaluate the impact of exercise on gut microbiota composition in individuals with obesity and T2D. The findings aim to provide robust scientific evidence supporting exercise as a non-pharmacological strategy for gut microbiota modulation and metabolic health improvement.

## 2 Methods

### 2.1 Protocol and registration

This systematic review was conducted following the Preferred Reporting Items for Systematic Reviews and Meta-Analyses (PRISMA) 2020 guidelines (Page et al., 2021) and has been prospectively registered in the PROSPERO database (Registration No. CRD420250653594). The complete PRISMA 2020 checklist is available in the Supplementary Table 1.

### 2.2 Information sources

A comprehensive literature search was performed across PubMed, Embase, Web of Science, and the Cochrane Library from their inception to February 17, 2025.

### 2.3 Search strategy

To ensure a systematic and comprehensive literature search, three thematic clusters of keywords were developed and combined: (1) gut microbiota–related terms (e.g., “gut microbiota,” “intestinal flora”); (2) exercise intervention–related terms (e.g., “exercise,” “physical activity,” “training”); and (3) target population terms (e.g., “obesity,” “type 2 diabetes”). The complete search strategy is detailed in Supplementary Table 2.

### 2.4 Selection process

All retrieved records were imported into EndNote software for deduplication. Literature screening was then performed in two stages. In the first stage, two independent reviewers (Qian and Pan) screened titles and abstracts based on a pre-specified PICO framework. Studies were categorized as “relevant,” “irrelevant,” or “uncertain” according to the predefined inclusion and exclusion criteria. Articles deemed “irrelevant” were excluded, with reasons documented. Those classified as “relevant” or “uncertain” proceeded to full-text review. In the second stage, two reviewers independently assessed the full texts to determine final eligibility. Disagreements were resolved through discussion with a third independent reviewer (Lin), who made the final decision if consensus could not be reached. Screening outcomes, including the number of included studies and reasons for exclusion, were recorded.

### 2.5 Inclusion and exclusion criteria

#### 2.5.1 Inclusion criteria

The eligibility criteria were established based on the PICOS framework as recommended by the Cochrane Handbook, and included the following components: P (Population): Studies involving individuals with obesity or type 2 diabetes (T2D); I (Intervention): Interventions limited to various forms of physical activity or exercise, including but not limited to aerobic or resistance training; C (Comparator): Studies with a clearly defined control group, such as no exercise or maintenance of habitual lifestyle; O (Outcomes): Studies that assessed gut microbiota composition using 16S rRNA gene sequencing or shotgun metagenomic sequencing, and reported microbial diversity (e.g., α-diversity or β-diversity) or taxonomic abundance; S (Study design): Controlled trials (including randomized and non-randomized controlled trials) with clearly defined intervention and control groups to ensure comparability of intervention effects.

#### 2.5.2 Exclusion criteria

Studies were excluded if they met any of the following conditions: Non-human studies (animal models). Non-original research, including review articles, case reports, conference abstracts, and letters. Studies not published in English.

### 2.6 Data collection process

Data extraction was conducted independently by two reviewers (Cheng and Pan) using a predesigned standardized extraction form. Discrepancies were resolved through discussion with a third reviewer (Lin) to reach consensus. The following key information was extracted:

A.    Study characteristics: First author’s name, year of publication, and country or region where the study was conducted.
B.    Participant characteristics: Sample size and group allocation, mean age and gender distribution, and participants’ health status (e.g., obesity, T2D, or other metabolic disorders).
C.    Intervention details: Type of intervention (e.g., aerobic exercise, resistance training), frequency, intensity, duration, total intervention period, and comparator condition (e.g., no intervention or usual care).
D.    Outcome measures: Gut microbiota outcomes including diversity (α- and β-diversity), changes in abundance, and other functional indicators.

For missing or unpublished data, study authors were contacted via email to obtain the necessary information. For data presented only in graphical format, numeric values were extracted using WebPlotDigitizer (v.4.4)^1^ or the built-in measuring tool in Adobe Acrobat, both of which have demonstrated high reliability and validity (Drevon et al., 2017). If essential data could not be obtained, the study was excluded from the analysis.

### 2.7 Data conversion

For studies that reported data as median and interquartile range (IQR), values were converted to mean (M) and standard deviation (SD) using an established online tool^2^ (Cumpston et al., 2019), This method has been widely applied in previous studies and validated for its reliability and accuracy (Nikolova et al., 2021). In cases where the reported data were substantially skewed, the conventional method for converting median and IQR to mean and SD might introduce bias. Therefore, to enhance the accuracy and robustness of the converted estimates, we applied alternative validated methods recommended by Luo et al. (2018) and Wan et al. (2014), which have been widely used for skewed or non-normally distributed data in meta-analyses.

### 2.8 Study risk of bias assessment

In this study, the methodological quality of the included studies was independently assessed by two reviewers using the risk of bias tools recommended in the Cochrane Handbook for Systematic Reviews of Interventions. For randomized controlled trials (RCTs), the Cochrane Risk of Bias 2.0 tool (RoB 2) was applied, which evaluates five key domains: random sequence generation, allocation concealment, blinding of participants and personnel, handling of incomplete outcome data, and selective outcome reporting (Sterne et al., 2019). For non-randomized controlled studies, the Risk of Bias in Non-randomized Studies of Interventions (ROBINS-I) tool was used (Sterne et al., 2016). This tool evaluates seven domains: confounding, selection of participants, classification of interventions, deviations from intended interventions, missing data, measurement of outcomes, and selection of the reported result. Each domain was rated as “low risk,” “some concerns,” or “high risk” of bias. Disagreements during the assessment process were resolved through discussion, and if necessary, adjudicated by a third independent reviewer.

Additionally, considering that some included studies lacked sufficient methodological detail in their reporting, we incorporated the Physiotherapy Evidence Database (PEDro) scale as a supplementary tool to enhance the comprehensiveness of the risk of bias evaluation (de Morton, 2009). The PEDro scale, widely used in assessing the methodological quality of intervention studies in the fields of physical therapy and exercise science, includes 10 criteria with a total score of 10. A score of ≥6 was considered high quality, 4–5 moderate quality, and ≤3 low quality.

### 2.9 Qualitative and quantitative data synthesis

Both qualitative (narrative) and quantitative (meta-analytic) syntheses were conducted to comprehensively evaluate the effects of exercise interventions on gut microbiota in individuals with obesity and type 2 diabetes (T2D). Quantitative meta-analyses were performed for outcomes with sufficient statistical information to compute effect sizes, such as α-diversity indices. In contrast, for β-diversity and changes in specific microbial taxa, where quantitative data were largely unavailable, a structured narrative synthesis was employed. Key information including intervention types, microbiological outcomes, and methodological characteristics was systematically integrated to enhance the comprehensiveness and interpretability of the evidence synthesis.

Quantitative analyses were conducted using Stata software (StataCorp, College Station, TX, USA). A random-effects model proposed by DerSimonian and Kacker (2007) was employed to calculate standardized mean differences (SMDs) with corresponding 95% confidence intervals (CIs) for comparing outcomes between intervention and control groups. Given the relatively small sample sizes for most outcomes, Hedges’ g correction was applied to reduce small-sample bias. The random-effects model was selected in light of the clinical and methodological heterogeneity observed among studies, providing a more conservative estimate by assuming that true effect sizes vary across studies rather than sharing a single underlying effect. This model accounts for both within-study and between-study variability and is therefore appropriate for the current meta-analysis given the diversity of exercise interventions included (Borenstein et al., 2010). Statistical heterogeneity was evaluated using the I^2^ statistic, with thresholds interpreted as follows: 0%–40%: might not be important; 30%–60%: may represent moderate heterogeneity; 50%–90%: may represent substantial heterogeneity; 75%–100%: considerable heterogeneity (Higgins et al., 2024). For outcomes with ≥10 included studies, potential publication bias was assessed using funnel plots and Egger’s regression test (Jin et al., 2015). To assess the robustness of the pooled effect estimates, a leave-one-out sensitivity analysis was also performed to evaluate the influence of individual studies on overall results.

To explore potential sources of heterogeneity and further evaluate effect modifiers of exercise interventions, we conducted subgroup and meta-regression analyses. Subgroup analyses were stratified by exercise type, intervention duration, and mean participant age, with between-group differences assessed using Cochran’s Q test, considering *P* < 0.1 as indicative of statistical significance (Nakagawa et al., 2017). In parallel, we performed random-effects meta-regression analyses examining variables such as country, mean participant age, exercise type, intervention duration, and sex to evaluate their contributions to the variability in effect sizes.

### 2.10 Certainty of evidence

The validity of each study was assessed in conjunction with quality ratings to support the interpretation of results. The Grading of Recommendations, Assessment, Development, and Evaluation (GRADE) framework (Schünemann et al., 2019) was utilized to evaluate the certainty of the evidence, categorizing it into high, moderate, low, or very low (Guyatt et al., 2011). Certainty assessment was independently performed by two reviewers, with disagreements resolved through discussion and, if necessary, arbitration by a third reviewer. The overall quality of evidence was graded based on the following criteria:

A.    Risk of Bias: If more than 50% of the total sample size in the included studies originated from trials with methodological concerns (e.g., one or more domains rated as “high risk” of bias), the certainty of evidence was downgraded by one level. If the primary outcome relied heavily on studies deemed to be at high risk of bias, a further downgrade was considered.
B.    Inconsistency: When substantial statistical heterogeneity was observed (I^2^ > 40%) without a plausible clinical or methodological explanation, the evidence was downgraded by one level. If heterogeneity was considerable (I^2^ > 75%) and accompanied by inconsistency in the direction or magnitude of effect estimates (e.g., low overlap in confidence intervals), the evidence was downgraded by two levels.
C.    Imprecision: If the total sample size was below 100 participants or if the 95% confidence interval crossed the line of no effect, the certainty was downgraded by one level. In cases where the confidence interval was excessively wide, making it difficult to determine the magnitude or direction of effect, an additional downgrade was considered (Vieira et al., 2022).
D.    Indirectness: The evidence was downgraded if the intervention was evaluated in populations or settings that differed meaningfully from the population of interest, or if indirect comparisons were used to infer the effects of the intervention.

## 3 Results

### 3.1 Study selection

**FIGURE 1:**
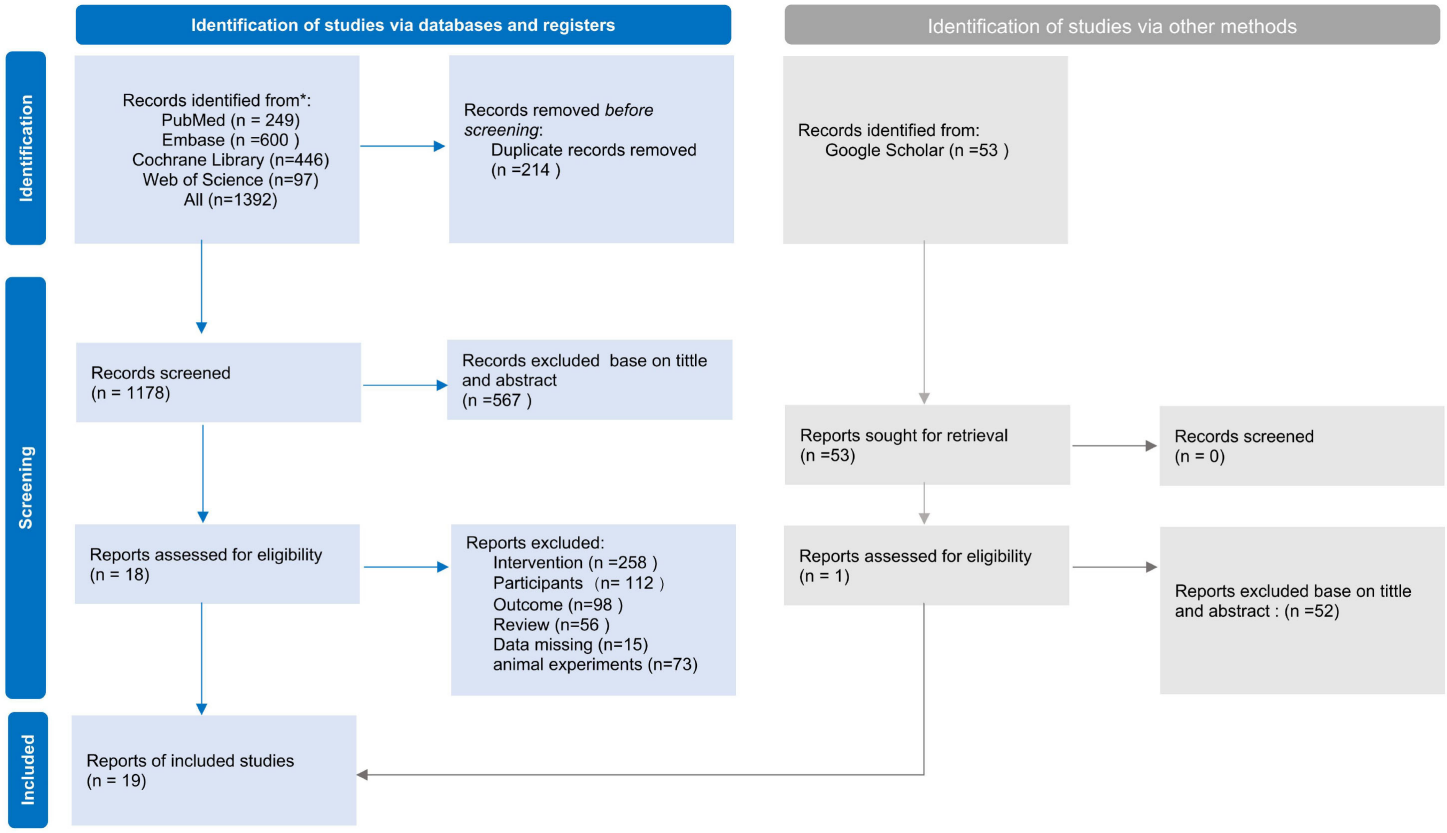
Preferred reporting items for systematic reviews and meta-analyses (PRISMA) flow diagram of the literature search and study selection process.

A total of 1,392 studies were identified through the database search. After removing 214 duplicates using EndNote, the remaining 1,179 studies were screened based on title and abstract. Following full-text evaluation, 19 studies met the eligibility criteria and were included in the systematic review. One study was excluded from the quantitative meta-analysis due to unavailable key outcome data, resulting in 18 studies being incorporated into the meta-analysis (Figure 1).

### 3.2 Characteristics of included studies

#### 3.2.1 Study characteristics

A total of 19 studies were included (Allen et al., 2018; Cronin et al., 2018; Guevara-Cruz et al., 2019; Kern et al., 2020; Cho, 2021; Cheng et al., 2022; Dupuit et al., 2022; Wei et al., 2022; Zhong et al., 2022; Beals et al., 2023; Hintikka et al., 2023; Lin et al., 2023; Torquati et al., 2023; Batitucci et al., 2024; Cullen et al., 2024; García-Gavilán et al., 2024; Lietzén et al., 2024; Nechalová et al., 2024; Wang et al., 2024), comprising 1,062 participants, 557 in the intervention group and 505 in the control group. These studies were conducted across multiple countries and regions, with the majority based in Europe (8 studies, including Denmark, Finland, and Slovakia) (Cronin et al., 2018; Kern et al., 2020; Wei et al., 2022; Hintikka et al., 2023; Cullen et al., 2024; García-Gavilán et al., 2024; Lietzén et al., 2024; Nechalová et al., 2024) and China (5 studies) (Cheng et al., 2022; Dupuit et al., 2022; Zhong et al., 2022; Lin et al., 2023; Wang et al., 2024). The remaining studies were conducted in the United States (2 studies) (Allen et al., 2018; Beals et al., 2023), Brazil (Batitucci et al., 2024), Australia (Torquati et al., 2023), South Korea (Cho, 2021), and Mexico (Guevara-Cruz et al., 2019) (each with one study). Regarding sex distribution, 13 studies included both male and female participants (Cronin et al., 2018; Kern et al., 2020; Wei et al., 2022; Hintikka et al., 2023; Cullen et al., 2024; García-Gavilán et al., 2024; Lietzén et al., 2024; Nechalová et al., 2024), while 4 studies focused exclusively on female participants (Dupuit et al., 2022; Zhong et al., 2022; Batitucci et al., 2024), and 2 studies included only male participants (Cho, 2021). In terms of participant health status, 12 studies focused on individuals with obesity (Allen et al., 2018; Cronin et al., 2018; Guevara-Cruz et al., 2019; Kern et al., 2020; Cho, 2021; Dupuit et al., 2022; Hintikka et al., 2023; Lin et al., 2023; Batitucci et al., 2024; Cullen et al., 2024; Lietzén et al., 2024; Nechalová et al., 2024), while 5 studies included participants with both obesity and T2D (Wei et al., 2022; Beals et al., 2023; García-Gavilán et al., 2024; Wang et al., 2024). Additionally, some studies investigated individuals with obesity in conjunction with other chronic diseases. The mean age of participants ranged from 18 to 75 years, covering young adults, middle-aged individuals, and older adults. Detailed information on the included studies is provided in Supplementary Table 3.

#### 3.2.2 Dietary and medication control

All included studies implemented some degree of dietary and medication control (see Supplementary Table 7 for details). On the dietary level, the most common approach was dietary records or questionnaire-based monitoring (11 studies), followed by nutritionist-led counseling or dietary education (7 studies), and energy/macronutrient-based allocation or restriction strategies (7 studies). Six studies required participants to maintain their habitual diet, and one study adopted app-based/digital reminders and logging. With respect to medication, most studies minimized confounding by excluding recent antibiotic/probiotic use (11 studies) and documented medication or health information to enhance traceability (10 studies). Only a few studies explicitly required stable medication regimens during the intervention period (1 study) or implemented a standardized medication management algorithm (1 study). Overall, the included studies applied varying degrees of control and monitoring for diet and medication, but specific strategies and reporting practices differed. We have provided a detailed, study-by-study description of these control methods and their implementation in Supplementary Table 7.

### 3.3 Meta-analysis

#### 3.3.1 Effect of exercise on alpha diversity

The majority of the included studies reported changes in alpha diversity following exercise interventions. Given the potential baseline differences in gut microbiota composition between individuals with obesity and those with T2D, the results were analyzed separately for each group to ensure a more accurate and meaningful interpretation.

**FIGURE 2:**
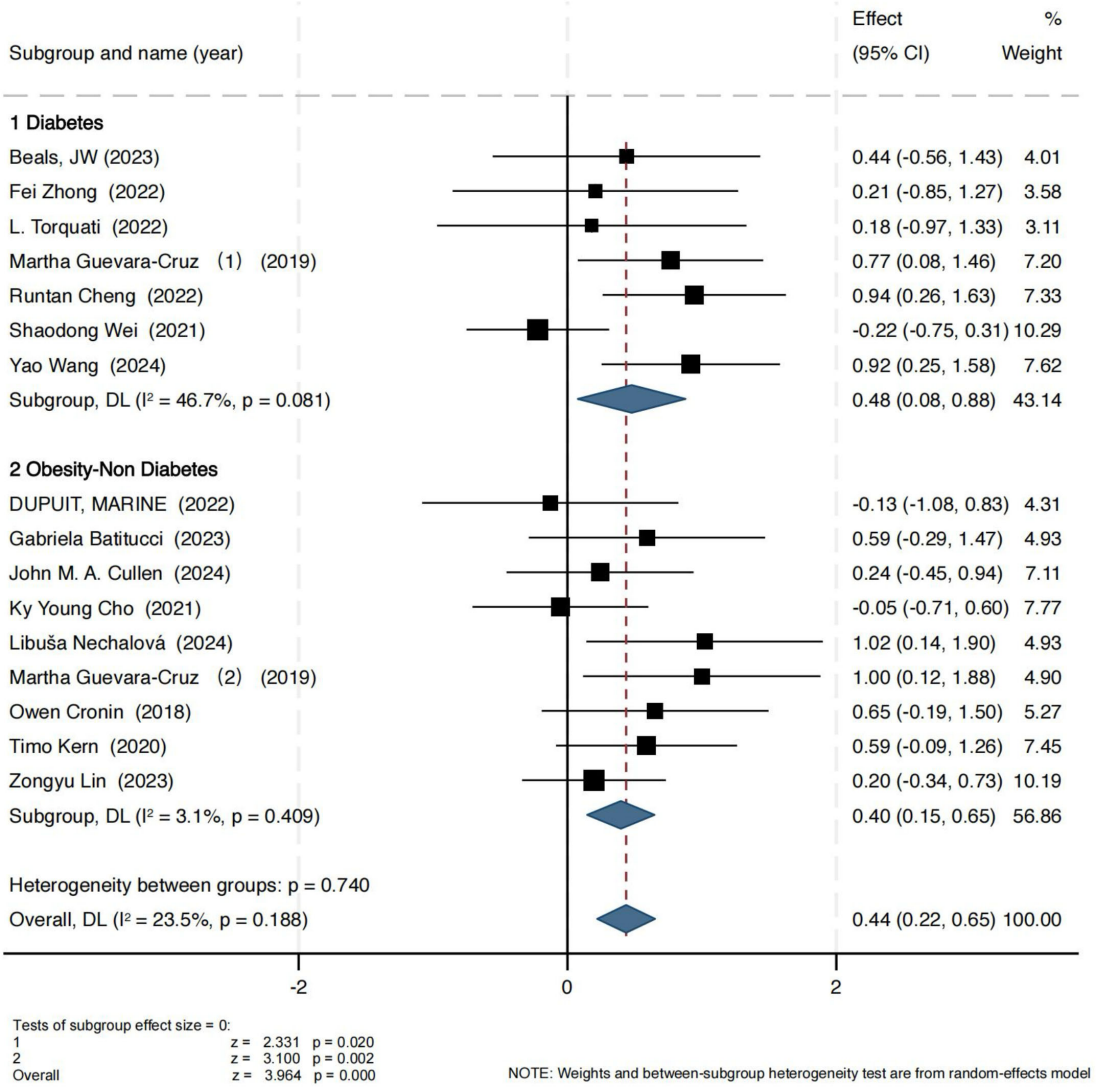
Forest plot of the main effect on the Shannon index.

For the Shannon index, the results are presented in Figure 2. In the obesity group, the pooled effect size showed a standardized mean difference (SMD) of 0.40 (95% CI: 0.15, 0.65), with a Z-score of 3.10 (*P* = 0.002, I^2^ = 3.1%), indicating low heterogeneity and strong consistency across studies. In the T2D group, exercise also led to a statistically significant improvement in the Shannon index (SMD = 0.48, 95% CI: 0.08, 0.88), with a Z-score of 2.44 (*P* = 0.02, I^2^ = 46.7%), suggesting moderate heterogeneity.

**FIGURE 3:**
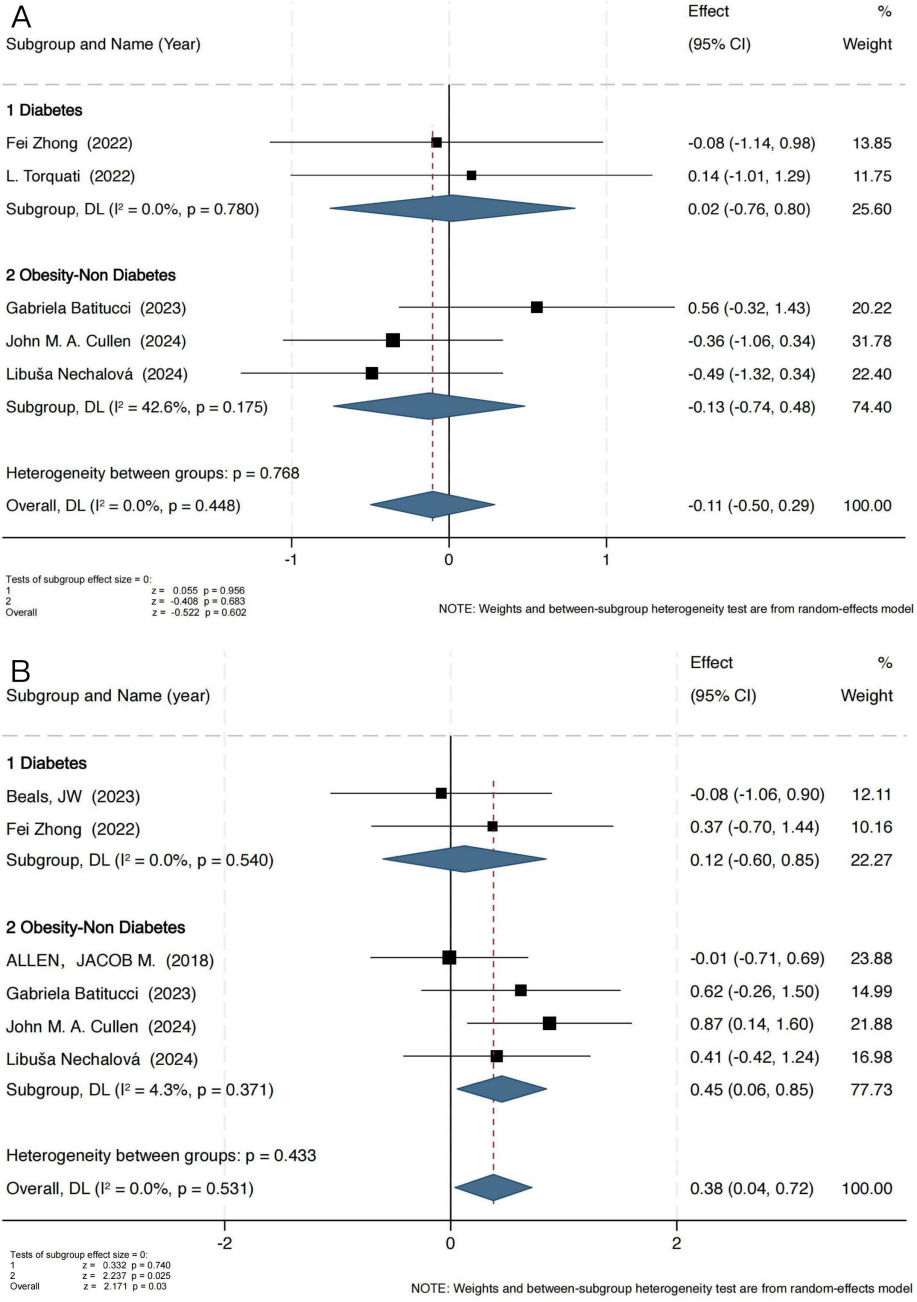
Forest plot of the main effect on the Simpson **(A)** and Chao1 **(B)** index.

The Simpson index results indicated that exercise intervention did not exert a significant effect on microbial evenness (Figure 3A). In the obesity group, the pooled standardized mean difference (SMD) was −0.13 (95% CI: −0.74 to 0.48), with a Z-score of −0.408 (*P* = 0.683, I^2^ = 42.6%), indicating moderate heterogeneity. In the T2D group, the SMD was 0.02 (95% CI: −0.76 to 0.80), with a Z-score of 0.055 (*P* = 0.956, I^2^ = 0%), demonstrating high consistency across studies (I^2^ = 0%).

These findings suggest that exercise has a limited impact on microbial evenness, potentially exerting a greater influence on microbial diversity and richness. To further investigate this, an additional analysis was conducted using the Chao1 index (Figure 3B), which specifically reflects microbial richness. Notably, the Chao1 index analysis revealed a significant positive effect of exercise intervention in the obesity group, with an SMD of 0.45 (95% CI: 0.06 to 0.85), a Z-score of 2.237 (*P* = 0.025, I^2^ = 4.3%), indicating low heterogeneity. However, in the T2D group, no significant change was observed (SMD = 0.12, 95% CI: −0.60 to 0.85), with a Z-score of 0.332 (*P* = 0.74, I^2^ = 0%).

**FIGURE 4:**
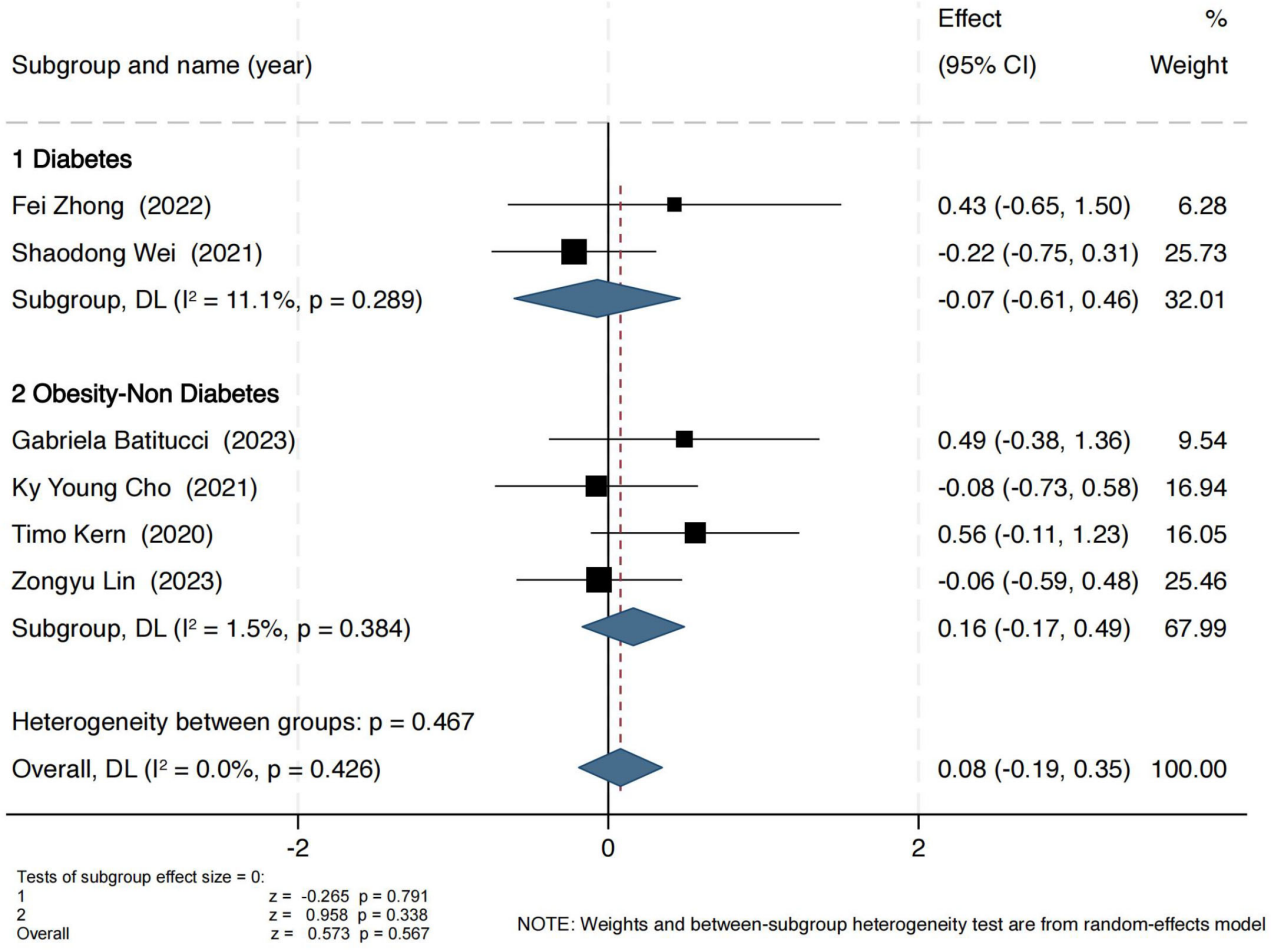
Forest plot of the main effect on the Observed OUTs index.

Observed OTUs were used to assess the number of observed microbial species. As shown in Figure 4, exercise interventions did not yield significant effects in either group. In the obesity group, the pooled standardized mean difference (SMD) was 0.16 (95% CI: −0.17 to 0.49), with a Z-score of 0.96 (*P* = 0.34, I^2^ = 2%), indicating low heterogeneity. In the T2D group, the SMD was −0.07 (95% CI: −0.61 to 0.46), with a Z-score of 0.26 (*P* = 0.79, I^2^ = 11%), also suggesting no significant intervention effect.

It should be noted that, because most of the included studies did not report Chao1 and Observed OTUs, the number of studies available for this analysis was limited (Chao1: *n* = 7; Simpson: *n* = 7; Observed OTUs: *n* = 6), which may have partially reduced the statistical power and increased the uncertainty of the results. Therefore, these findings should be interpreted with caution.

#### 3.3.2 Subgroup analysis

Given that different types of exercise may exert varying effects on gut microbiota (GM) and that GM composition differs significantly across age groups, a subgroup analysis was performed to further refine the impact of exercise interventions. Due to limited data availability, this analysis primarily focused on differences in the Shannon index.

**FIGURE 5:**
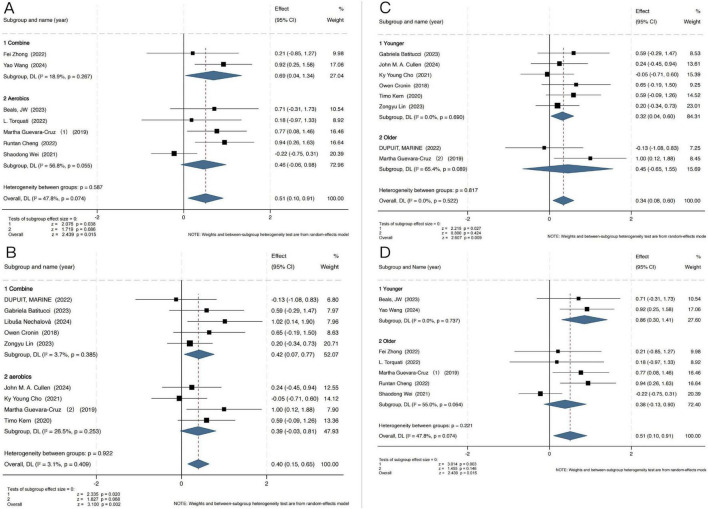
Effects of different exercise modalities on the Shannon index in individuals with T2D **(A)** and Obesity **(B)**; effects of exercise on the Shannon index across different age groups in individuals with obesity **(C)** and T2D **(D)**.

Given the predominant exercise modalities adopted in the included studies, we categorized interventions into aerobic-only and multi-component exercise programs (Combined exercise). As shown in Figure 5A, in individuals with T2D, Combined exercise had a more pronounced effect on gut microbiota (GM) diversity (SMD = 0.69, 95% CI [0.04, 1.34], *P* = 0.04). A similar pattern was observed in the obesity group (Figure 5B), where combined exercise also yielded significant improvements (SMD = 0.42, 95% CI [0.07, 0.77], *P* = 0.02).

To further explore potential moderators, subgroup analyses were conducted based on participant age, stratified into younger (<50 years) and older (≥50 years) groups. The results demonstrated a more substantial effect of exercise on GM diversity in younger individuals, both in the obesity subgroup (Figure 5C) (SMD = 0.32, 95% CI [0.04, 0.60], *P* = 0.03) and in the T2D subgroup (Figure 5D) (SMD = 0.86, 95% CI [0.30, 1.41], *P* = 0.003). In the subgroup analysis based on intervention duration (Supplementary Figure 1), the included studies were categorized into short-term interventions (<12 weeks) and long-term interventions (≥12 weeks). The results showed that short-term interventions yielded more pronounced effects on improving gut microbiota diversity (obesity: SMD = 0.41, 95% CI [0.05, 0.77], *P* = 0.027; T2D: SMD = 0.52, 95% CI [0.02, 1.03], *P* = 0.041). This finding suggests that the duration of exercise may be one of the factors influencing intervention effectiveness. It should be noted, however, that although heterogeneity was low, the results of subgroup analyses must be interpreted with caution due to the limited number of available studies, which may have influenced the stability of the findings.

### 3.4 Qualitative synthesis of findings across included studies

#### 3.4.1 Beta diversity

A total of 17 studies assessed the impact of exercise interventions on gut microbiota beta diversity in individuals with obesity and type 2 diabetes (T2D). The main findings and characteristics of the included studies were summarized in Supplementary Table 4. The majority of included studies employed indices such as Unweighted UniFrac, Weighted UniFrac, and Bray-Curtis, in conjunction with principal coordinate analysis (PCoA) and permutational multivariate analysis of variance (PERMANOVA) to evaluate differences in microbial community composition betwee n intervention and control groups. Most studies demonstrated a positive effect of exercise on gut microbiota structure. For instance, significant between-group differences were reported in the studies by Dupuit et al. (2022), Lin et al. (2023), Cheng et al. (2022), and Cronin et al. (2018) based on Weighted UniFrac and Bray-Curtis metrics, suggesting that exercise interventions may effectively alter the microbial community composition. Similar trends were observed in studies focusing on T2D populations, such as those conducted by Wei et al. (2022) and Torquati et al. (2023), further confirming the beneficial impact of exercise on gut microbiota composition in T2D patients. However, inconsistencies in beta diversity findings were also noted. Certain studies, such as those by Cullen et al. (2024) and Batitucci et al. (2024), did not observe significant differences in the reported beta diversity metrics. Moreover, some studies revealed discrepancies between different analytical methods (e.g., Weighted vs. Unweighted UniFrac), highlighting the inherent complexity and methodological variability in beta diversity assessment. Overall, despite some heterogeneity in results, exercise interventions appear to exert a measurable influence on gut microbiota beta diversity in both individuals with obesity and those with T2D.

#### 3.4.2 Potential regulatory effects of exercise on gut microbial function and metabolites

Multiple studies consistently demonstrate that exercise interventions are associated with an enrichment of functional clusters involved in butyrate production (Supplementary Table 6). Representative taxa include members of the *Roseburia* genus, *Faecalibacterium prausnitzii*, and *Eubacterium coprostanoligenes*, with such changes reported in studies by Allen et al. (2018), Cullen et al. (2024), and Guevara-Cruz et al. (2019). These microbes may contribute to maintaining intestinal barrier integrity, reducing low-grade inflammation, and improving insulin sensitivity through the synthesis of butyrate. Concurrently, a marked increase in the relative abundance of *Akkermansia muciniphila* has been observed in studies by Hintikka et al. (2023), Nechalová et al. (2024), and Guevara-Cruz et al. (2019), suggesting that exercise may enhance host energy homeostasis by modulating mucin-layer metabolism. In contrast, changes in the Firmicutes/Bacteroidetes (F/B) ratio at the phylum level are inconsistent across studies: some report an increase in Firmicutes accompanied by a decrease in Bacteroidetes (Cho, 2021; Lietzén et al., 2024), whereas others find no significant trend (Lin et al., 2023). This variability indicates that interpretation of this index is highly dependent on baseline microbiota composition, dietary control, and the specifics of the exercise prescription. Moreover, taxa associated with metabolic risk exhibit pronounced context dependence. For example, *Prevotella copri* (a species within the *Prevotella* genus) increased significantly following exercise in the study by Cronin et al. (2018) but decreased in studies by Zhong et al. (2022) and Guevara-Cruz et al. (2019). Similarly, *Bacteroides* abundance increased in some studies (Wei et al., 2022; Guevara-Cruz et al., 2019) and decreased in others (Allen et al., 2018; Cho, 2021). These discrepancies suggest that their metabolic relevance is influenced by dietary composition, energy balance, and host-specific factors, cautioning against simplistic binary interpretations. Additionally, a few studies have explored the gut mycobiome. For instance, (Wang et al., 2024) reported increased abundance of *Verticillium* and *Sarocladium*, which correlated with improvements in HOMA-IR and blood lipid profiles, suggesting that exercise-induced modulation of fungal communities may also contribute to host metabolic regulation, although the current evidence remains extremely limited.

Regarding metabolic metabolites, approximately half of the studies observed an increase in total short-chain fatty acids (SCFAs) or their components (acetate, propionate, and butyrate) following exercise (Allen et al., 2018; Guevara-Cruz et al., 2019; Cheng et al., 2022), showing a structural–functional correspondence with the enrichment of butyrate-producing taxa. However, some studies reported discrepancies in the direction of changes between serum and fecal SCFA levels (Hintikka et al., 2023; Nechalová et al., 2024), or a decline after training cessation (Allen et al., 2018), suggesting that differences in biological matrices and sampling time may be key influencing factors. In terms of bile acid metabolism, (Lin et al., 2023; García-Gavilán et al., 2024; Nechalová et al., 2024) documented alterations in bile acid profiles (e.g., shifts in secondary-to-primary ratios) that paralleled changes in bile salt hydrolase (BSH)-producing taxa, implicating the FXR/TGR5 signaling pathway as a potential mediator of the “exercise–microbiota–host metabolism” axis. For amino acid and lipid metabolism, (Hintikka et al., 2023) and (Nechalová et al., 2024) reported decreases in multiple amino acids accompanied by significant remodeling of lipid metabolic networks, while (García-Gavilán et al., 2024) also observed downregulation of sphingolipid and ceramide metabolism. With respect to endotoxin burden, (Cho, 2021) and (García-Gavilán et al., 2024) reported reductions in LPS or LBP levels, consistent with improvements in inflammatory markers, supporting the notion that exercise may attenuate low-grade inflammation by reducing metabolic endotoxemia. Trimethylamine N-oxide (TMAO) showed a decreasing trend in Cronin et al. (2018), although overall evidence remains limited.

Several studies have further evaluated the associations between gut microbiota or metabolites and clinical phenotypes. The abundance of *Akkermansia muciniphila* has been negatively correlated with reductions in body weight and BMI (Hintikka et al., 2023; Nechalová et al., 2024). Increases in *Roseburia* and *Faecalibacterium prausnitzii* were accompanied by decreases in fat mass, improvements in HDL-C, and optimization of HOMA-IR (Guevara-Cruz et al., 2019; Cullen et al., 2024). Subgroups of *Prevotella* and *Bacteroides* in different studies were also linked to glycemic or lipid parameters (Cronin et al., 2018; Zhong et al., 2022). Regarding the mycobiome, (Wang et al., 2024) reported that increases in *Verticillium* and *Sarocladium* were associated with decreases in fasting insulin and LDL-C. Another study (Dupuit et al., 2022) found that the abundance of Bifidobacteriaceae correlated positively with fat mass and negatively with muscle mass and HDL-C, whereas the Paraprevotellaceae and Prevotellaceae families displayed the opposite pattern. Although these findings suggest potential directional relevance, the limited sample sizes and insufficient statistical control preclude definitive conclusions regarding causality.

In summary, the most consistent signals observed following exercise include: (1) increased abundance of butyrate-producing taxa and *Akkermansia*, (2) elevations in SCFA levels in some studies with subsequent declines after training cessation, (3) reductions in endotoxin burden, and (4) alterations in bile acid and amino acid/lipid metabolism. These changes align closely with enhanced gut barrier function, attenuation of inflammation, improved insulin sensitivity, and optimized lipid homeostasis. However, given the heterogeneity across studies and the limited strength of current evidence, standardized studies integrating metagenomics, metabolomics, and longitudinal sampling are urgently needed to further validate the role of the “microbiota–metabolism axis” in the management of obesity and T2D.

### 3.5 Risk of bias

#### 3.5.1 Risk of bias and quality of methods

**FIGURE 6:**
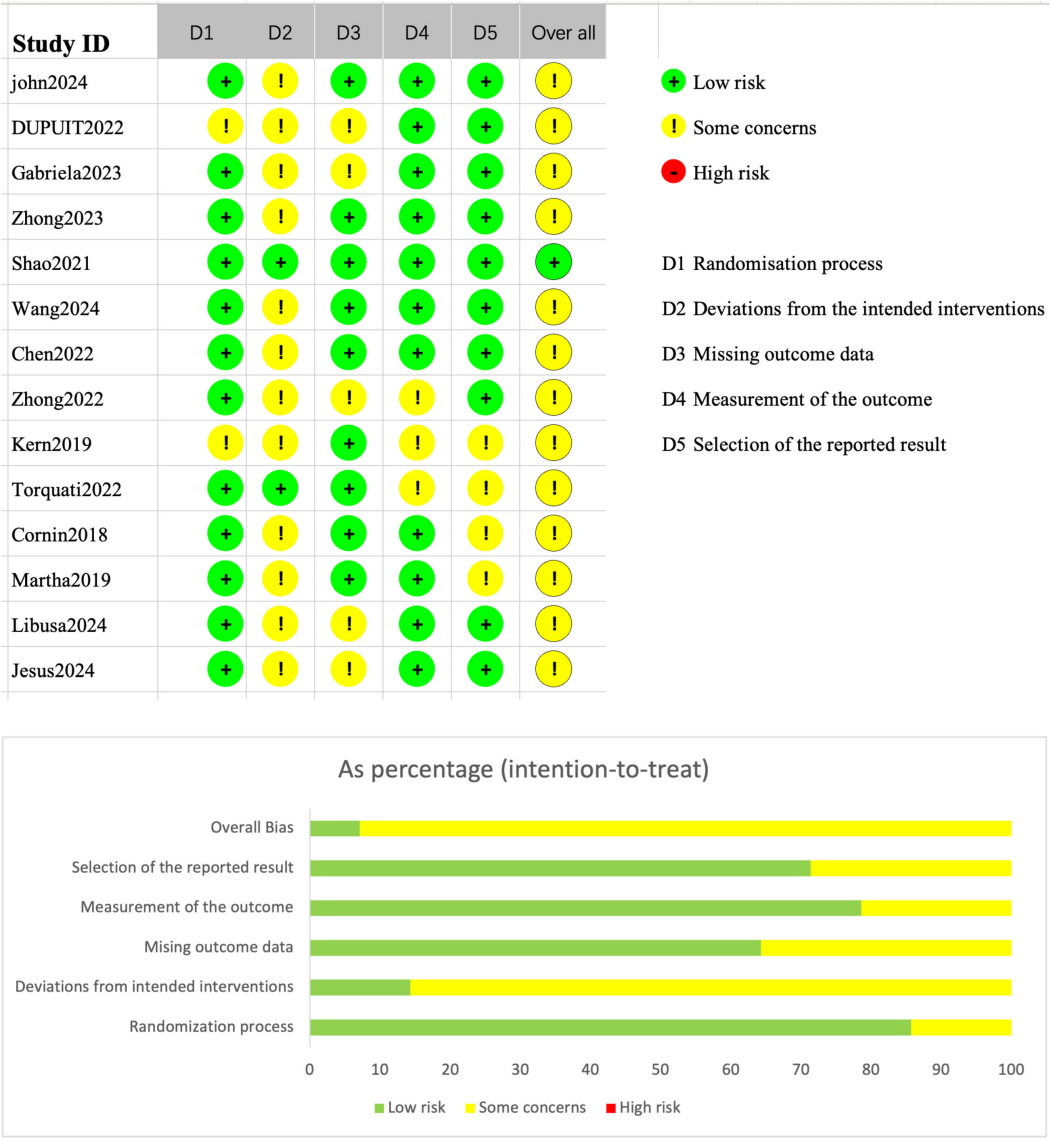
Cochrane Risk of Bias 2.0 tool (RoB 2) results.

The risk of bias for the included randomized controlled trials (RCTs) was assessed using the Cochrane Risk of Bias 2 (RoB 2) tool, as presented in Figure 6. Most studies were rated as low risk across multiple domains, and none of the RCTs were judged to have an overall “high risk” of bias. Specifically, the domains of the randomization process and missing outcome data generally demonstrated good methodological quality. However, some concerns were identified in the domain of deviations from intended interventions, primarily due to the absence of blinding or protocol deviations in several studies. In addition, a few studies raised minor concerns regarding the selection of the reported results.

Figure 6 also illustrates the proportional distribution of risk ratings across different domains: approximately 70% of the studies were rated as “some concerns” in the deviations from intended interventions domain, whereas more than 80% of the studies were rated as low risk for outcome data completeness and outcome measurement. As a result, the majority of RCTs were categorized as having “some concerns” overall, with only one or two studies rated as low risk across all domains and thus classified as having an “overall low risk” of bias.

For non-randomized intervention studies, methodological quality was assessed using the ROBINS-I tool. As shown in Table 1, the overall risk of bias was judged to be moderate, primarily due to inadequate control of confounding factors. All included studies were rated as having a “moderate risk” of bias in the domain of confounding. Additionally, one study was rated as having a moderate risk in the “deviations from intended interventions” domain due to inconsistencies in intervention implementation in the absence of random allocation. The remaining domains were generally assessed as low risk.

**TABLE 1:** Risk of bias in non-randomized studies of interventions (ROBINS-1) results.

ROBINS-1 assessment of non-randomized trials								
Study	Bias in selection of participants	Bias in classification of interventions	Bias due to deviations	Bias due to missing data	Bias in measurement	Bias in reported result	Bias due to confounding	Overall risk
Cho, 2021	Low	Low	Low	Low	Low	Low	Moderate^1^	Moderate
Hintikka et al., 2023	Low	Low	Moderate^2^	Low	Low	Low	Moderate^3^	Moderate
Allen et al., 2018	Low	Low	Low	Low	Low	Low	Moderate^4^	Moderate
Lietzén et al., 2024	Low	Low	Low	Low	Low	Low	Moderate^5^	Moderate
Beals et al., 2023	Low	Low	Low	Low	Low	Low	Moderate^6^	Moderate

^1^ Key variables such asdietary habits and physical activity were not statistically adjusted for, thus judged as moderate risk.

^2^ Control participants were instructed tomaintain habitual routines, adherencewas not objectively verified.

^3^ Key lifestyle factors such as diet and habitual activity were recorded butnot statistically adjusted for, posing moderate confounding risk.

^4^ Key confounders such as diet, physical activity, and sex ratio were not statistically adjusted for.

^5^ Dietary variation and quality of unsupervised exercise were not statistically adjusted, leading to moderate risk.

^6^ Despite concurrent recruitment with identical criteria, the absence of randomization or adjustment methods (e.g., propensity scores) introduces potential confounding from individual motivation or physical capacity. Key covariates were reported but not consistently adjusted.

The methodological quality of randomized controlled trials (RCTs) was further evaluated using the PEDro scale, with scores ranging from 4 to 7 out of a maximum of 10. Detailed PEDro scores are presented in Table 2. According to established criteria, a score of ≥6 indicates high quality, 4–5 denotes moderate quality, and ≤3 represents low quality. All RCTs included in this review scored ≥4. Approximately half of the studies were rated as high quality (≥6), while the remainder were considered of moderate quality.

**TABLE 2:** PEDpro results.

References	D1	D2	D3	D4	D5	D6	D7	D8	D9	D10	D11	Total
Cullen et al., 2024	Yes	1	0	1	0	0	1	1	0	1	1	6
Dupuit et al., 2022	Yes	1	0	0	0	0	0	1	1	1	1	5
Batitucci et al., 2024	Yes	1	1	1	0	0	0	1	1	1	1	7
Lin et al., 2023	Yes	1	1	1	0	0	0	1	1	1	1	7
Wei et al., 2022	Yes	1	1	1	0	0	0	1	1	1	1	7
Wang et al., 2024	Yes	1	0	1	0	0	0	1	0	1	1	5
Cheng et al., 2022	Yes	1	0	1	0	0	0	1	0	1	1	5
Zhong et al., 2022	Yes	1	1	0	0	0	0	1	0	1	1	5
Kern et al., 2020	Yes	1	0	1	0	0	0	1	1	1	1	6
Torquati et al., 2023	Yes	1	1	1	0	0	0	1	0	1	1	6
Cronin et al., 2018	Yes	1	1	1	0	0	0	1	0	1	1	6
Guevara-Cruz et al., 2019	Yes	1	1	1	0	0	0	1	1	1	1	7
Nechalová et al., 2024	Yes	1	0	1	0	0	0	0	0	1	1	4
García-Gavilán et al., 2024	Yes	1	1	1	0	0	0	1	0	1	1	6

Studies scoring ≥6 are considered high quality, those scoring 4–5 are considered moderate quality, and those scoring ≤3 are considered low quality. The PEDro scale consists of 11 items used to assess the methodological quality of randomized controlled trials. The first item (eligibility criteria were specified) pertains to external validity and is not included in the total score. The remaining 10 items are scored as either “yes” (1) or “no” (0), for a maximum total score of 10. The 11 items assessed are: 1. Eligibility criteria were specified (not included in the total score) 2. Subjects were randomly allocated to groups 3. Allocation was concealed 4. The groups were similar at baseline regarding the most important prognostic indicators 5. There was blinding of all subjects 6. There was blinding of all therapists who administered the therapy 7. There was blinding of all assessors who measured at least one key outcome 8. Measures of at least one key outcome were obtained from more than 85% of the subjects initially allocated to groups 9. All subjects for whom outcome measures were available received the treatment or control condition as allocated or, where this was not the case, data for at least one key outcome were analyzed by “intention to treat” 10. The results of between-group statistical comparisons are reported for at least one key outcome 11. The study provides both point measures and measures of variability for at least one key outcome All scoring was independently performed by two reviewers; any disagreements were resolved through discussion or by involving a third reviewer.

Most studies clearly reported eligibility criteria, employed random allocation with adequate concealment, had complete outcome data, and utilized appropriate statistical analyses. A common limitation across studies was the lack of blinding: the majority were unable to blind participants or therapists, and some did not blind outcome assessors, which may have influenced the PEDro scores. Nevertheless, nearly all studies conducted intention-to-treat analyses and provided point estimates with measures of variability, enhancing the robustness of the findings.

Taken together, the overall methodological quality of the included studies was moderate to high, with no critical threats to internal validity. However, caution is warranted when interpreting the results, particularly considering the limitations related to incomplete blinding and potential residual confounding.

#### 3.5.2 Publication bias

**FIGURE 7:**
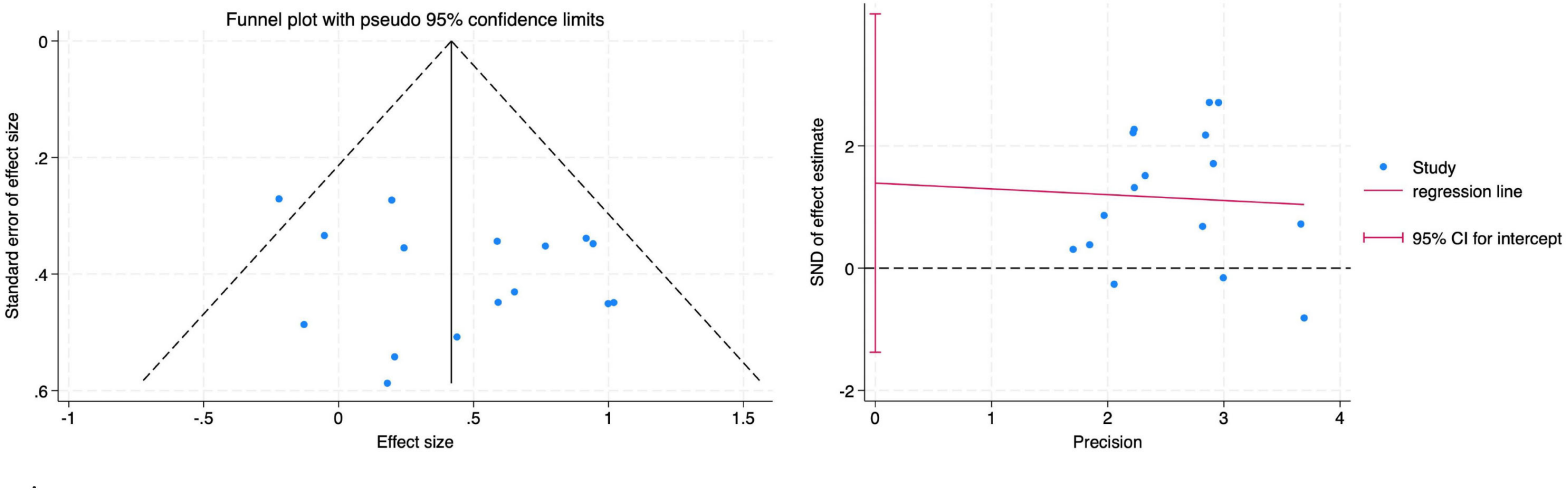
Funnel plot and Egger’s test for the main effect on the Shannon index.

The funnel plot did not indicate any apparent publication bias. This was further confirmed by Egger’s regression test, which showed no significant small-study effects across all diversity indices: Shannon index (Figure 7) (Root MSE = 1.137, *P* = 0.299).

#### 3.5.3 Sensitivity analyses

**FIGURE 8:**
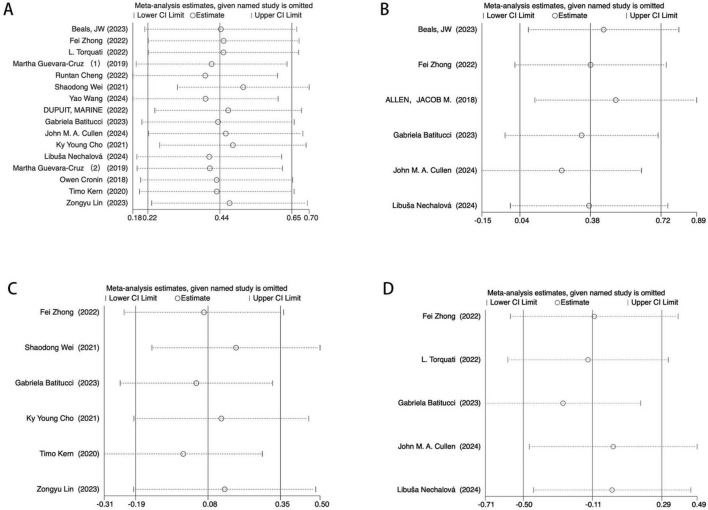
Egger’s test sensitivity analysis. **(A)** Sensitivity analysis of the main effect on the Shannon index. **(B)** Sensitivity analysis of the main effect on the Chao1 index index. **(C)** Sensitivity analysis of t main effect on the Observed OTUs. **(D)** Sensitivity analysis of the main effect on the Simpson index.

To further assess the robustness of the findings, a leave-one-out sensitivity analysis was performed on the primary effect sizes. The exclusion of any single study did not result in substantial changes in effect sizes for the Shannon (Figure 8A) and Simpson (Figure 8D) indices. Similarly, Chao1 (Figure 8B) and Observed OTUs (Figure 8C) remained unaffected under leave-one-out analysis, suggesting that the overall findings were not driven by any single study.

#### 3.5.4 Meta-regression

To further explore potential sources of heterogeneity, we performed meta-regression analyses incorporating variables such as country, mean age, exercise type, intervention duration, and sex proportion (see Supplementary Table 5 for detailed results). The analyses did not reveal any significant moderating effects of these variables on the effect sizes (*P* > 0.05). It should be noted, however, that the statistical power of the meta-regression may be limited due to the small number of available studies and incomplete reporting of certain covariates. Therefore, the meta-regression results in this study should be interpreted with caution and regarded as exploratory.

#### 3.5.5 GRADE

**FIGURE 9:**
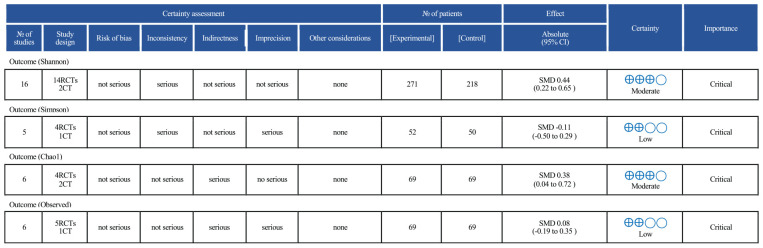
Grading of recommendations, assessment, development, and evaluation (GRADE) results.

The results of the GRADE assessment are presented in Figure 9. Among the four alpha diversity indices evaluated, two (Shannon and Chao1) were rated as having moderate certainty of evidence, while the other two (Simpson and Observed species) were rated as low certainty. The Shannon index was downgraded by one level due to statistical heterogeneity (I^2^ > 40%). The Simpson index was rated as low certainty owing to multiple concerns: a limited number of included studies (*n* = 5), small overall sample size, confidence intervals crossing the line of no effect, and the presence of both inconsistency and imprecision. Although the Chao1 index showed a statistically significant result, the certainty of evidence was rated as moderate due to potential residual confounding in some studies. The Observed OTUs index was downgraded due to moderate heterogeneity (I^2^ > 40%) and wide confidence intervals that included the null value, resulting in a low certainty rating.

## 4 Discussion

This meta-analysis aimed to evaluate the effects of exercise interventions on the gut microbiota (GM) in individuals with obesity and type 2 diabetes (T2D). A total of 19 study were included, and the findings indicate that exercise interventions can improve gut microbiota diversity to some extent in individuals with obesity and T2D, with particularly significant improvements observed in the Shannon and Chao1 indices. Additionally, our analysis demonstrated that combined exercise interventions exerted a more pronounced effect on gut microbiota diversity and function compared to single-mode interventions. Furthermore, age emerged as a key factor, with younger individuals exhibiting greater responsiveness to exercise interventions.

We aggregated effect sizes from 18 studies assessing alpha diversity, including the Shannon index, Simpson index, Chao1 index, and Observed OTUs. As a key measure of species richness and evenness within an ecosystem, alpha diversity is widely used to evaluate gut microbiota diversity (Gevers et al., 2012; Hagerty et al., 2020). Our findings demonstrated that exercise significantly improved the Shannon index, indicating an overall increase in alpha diversity. Previous studies have also highlighted the beneficial effects of exercise on gut microbiota. For instance, Min et al. (2024) reviewed 19 studies and found that exercise significantly enhanced gut microbiota diversity in adults. Our meta-analysis further substantiates the effectiveness of exercise interventions in both obese and T2D populations, reinforcing their potential value in managing these conditions. Evidence suggests that alterations in gut microbiota contribute to the development of obesity and T2D. For example, Zheng et al. (2022) analyzed 28 studies and demonstrated that exercise-induced modifications in gut microbiota were associated with weight reduction in obese individuals, which may be attributed to the enhanced microbial diversity facilitated by exercise. Similarly, Cullen et al. (2024) reported that 6 weeks of resistance training significantly increased alpha diversity in obese individuals while simultaneously reducing metabolic risk indicators. Current evidence indicates that exercise influences the progression of obesity and T2D by modulating gut microbiota composition. One proposed physiological mechanism is that exercise may regulate gut microbiota by promoting actin release, increasing gut motility, or stimulating the secretion of neurotransmitters and hormones (Aya et al., 2021). Notably, previous systematic reviews have confirmed that exercise exerts its effects on gut microbiota through multiple mechanisms, including variations in exercise intensity and duration, as well as differences between diseased and healthy populations (Mailing et al., 2019). Therefore, further well-designed studies are warranted to elucidate the underlying mechanisms. Additionally, we did not observe significant changes in the Simpson index or Observed OTUs, a finding consistent with previous studies. This suggests that while exercise may have a limited impact on microbial evenness, it likely exerts a more pronounced influence on species richness.

Interestingly, we observed a significant increase in the Chao1 index among individuals with obesity, whereas no significant change was found in the T2D group. Similar to the Shannon index, the Chao1 index is used to assess gut microbiota (GM) richness; however, unlike Shannon, Chao1 specifically emphasizes the abundance of rare microbial species (Schoch et al., 2022). This finding suggests that exercise may enhance the presence of less prevalent microbial species in individuals with obesity. The absence of a similar effect in the T2D group may be attributed to baseline differences in gut microbiota composition (Crudele et al., 2023). Although obesity and T2D are closely related, individuals with T2D often experience more complex immune dysregulation and tissue damage due to persistent hyperglycemia and elevated free fatty acids. These factors contribute to a higher prevalence of gut mucosal barrier impairment (Chong et al., 2025), which may hinder the colonization and proliferation of certain microbial species, thereby limiting improvements in microbial diversity (Yang et al., 2021). Consequently, the benefits of exercise interventions may vary across different disease populations. It is also important to note that the Chao1 index was not reported in most of the included studies, resulting in a limited dataset for this meta-analysis. This constraint may have influenced the results and thus warrants a cautious interpretation of the findings.

The benefits of exercise-induced gut microbiota modulation remain a topic of debate, primarily due to variations in exercise modalities and population-specific responses. While most studies have reported significant benefits, some have found no substantial effects. For instance, Batitucci et al. (2024) observed that high-intensity interval training (HIIT) did not significantly alter gut microbiota composition in obese women. This finding highlights the need for a more precise evaluation of exercise effects, considering both population characteristics and exercise types. Our subgroup analysis revealed that, compared to aerobic exercise alone, combined exercise interventions exerted a more pronounced effect on improving the Shannon index in both the obesity group (SMD = 0.42, *P* = 0.02) and the T2D group (SMD = 0.69, *P* = 0.04). Notably, no study has yet systematically compared the effectiveness of different exercise modalities on gut microbiota. While Aragón-Vela et al. (2021) suggested in a systematic review that a combination of resistance and aerobic training may reduce body fat and enhance gut microbiota diversity, their analysis did not quantify the effect sizes. Our study builds upon this by integrating recent and comprehensive evidence and providing a quantitative meta-analysis of the effects of aerobic and combined exercise on gut microbiota. Additionally, our findings indicate that exercise exerts a more pronounced impact on younger populations (<50 years), a trend observed in both individuals with obesity and those with T2D. This contrasts with the conclusions of Min et al. (2024) whose meta-analysis included a broader population encompassing healthy individuals, patients with chronic diseases, and athletes, potentially accounting for the discrepancies in findings. These results underscore the notion that exercise-induced gut microbiota alterations may differ significantly between healthy and diseased populations. Moreover, age plays a pivotal role in shaping gut microbiota composition. Viviana et al. conducted a systematic review of 15 studies (Aya et al., 2023) and reported that physical activity (PA) did not significantly impact gut microbiota diversity (both alpha and beta diversity) in older adults, a finding consistent with our meta-analysis. One possible explanation is that gut microbiota composition naturally changes with age, characterized by reduced microbial diversity and increased interindividual variability (Ling et al., 2022). Research suggests that Bacteroidetes levels tend to be higher in older adults, whereas Firmicutes are more prevalent in younger individuals (Mariat et al., 2009). As aging progresses, the gut microbiota undergoes substantial shifts, transitioning from a balanced microbial community to a dysbiotic state, often marked by an increased abundance of pathogenic bacteria. This shift may contribute to the diminished responsiveness of older populations to exercise interventions. Regarding exercise duration, our subgroup analysis indicated that short-term interventions (<12 weeks) were more likely to elicit improvements in gut microbiota diversity compared with long-term interventions (≥12 weeks). Similar findings have been reported in previous studies, where aerobic training induced detectable microbial alterations within as little as 2 weeks, with a relatively stable new composition emerging by approximately 6–8 weeks; by contrast, prolonging the intervention duration does not necessarily produce a linear amplification effect but may instead reach a “plateau phase” (Boytar et al., 2023). Furthermore, human intervention studies have observed that microbial and metabolite (e.g., SCFAs) changes may partially or completely revert after training cessation, highlighting that “continuous stimulation” is essential for maintaining these effects and that sampling time points and follow-up duration can markedly influence observations from long-term interventions (Allen et al., 2018). Therefore, prolonged training may not consistently yield concordant “dose–response” benefits, potentially because chronic stress under certain conditions may increase intestinal permeability and disrupt barrier integrity, thereby offsetting some of the beneficial signals (Munukka et al., 2018; Ortiz-Alvarez et al., 2020). Hence, exercise duration represents a critical determinant of adaptive gut microbiota remodeling. Elucidating the nonlinear dose–response relationship of exercise duration on gut microbiota, and clarifying the dynamic interplay among “duration–intensity–effect,” could provide more direct evidence for establishing standardized exercise prescriptions. It is important to acknowledge that although our subgroup analyses identified differences in exercise regimens as potential influencing factors, subgroup analyses alone cannot serve as direct evidence of underlying biological processes and should be interpreted as exploratory, aimed primarily at illustrating trends and generating hypotheses (Koch et al., 2023).

In addition to analyzing alpha diversity, we conducted a qualitative synthesis of beta diversity and taxonomic gut microbiota. The majority of the included studies reported significant post-intervention differences in gut microbiota composition between the experimental and control groups. These differences were primarily identified through principal coordinate analysis (PCoA) and permutational multivariate analysis of variance (PERMANOVA), both of which demonstrated clear group separation. From a metabolic health perspective, such structural modifications suggest that exercise may partially reshape the gut microbial ecosystem, potentially steering it toward a more metabolically favorable state for the host (Shahar et al., 2020). However, as none of the included studies incorporated a healthy control group, it remains unclear whether these alterations necessarily confer health benefits. Moreover, methodological inconsistencies in data analysis substantially influenced the results, thereby limiting the generalizability of our findings (Song et al., 2018). Therefore, while our study identifies a potential trend, definitive conclusions cannot yet be drawn. Regarding taxonomic gut microbiota, nearly all included studies reported an increase in beneficial bacterial taxa following exercise. Our key findings can be summarized into three main aspects. First, exercise was associated with an increased relative abundance of butyrate-producing bacteria. Butyrate plays a crucial role in glucose homeostasis and insulin sensitivity by binding to G-protein-coupled receptors (GPCRs), such as FFAR2/GPR43, on intestinal endocrine cells, thereby stimulating the secretion of glucagon-like peptide-1 (GLP-1) (Tolhurst et al., 2012; Greenhill, 2020). These findings suggest that exercise-induced gut microbiota alterations may contribute to the management of obesity and T2D through this pathway. Second, exercise appeared to modulate the Firmicutes-to-Bacteroidetes (F/B) ratio, a key microbial marker often associated with obesity. A higher F/B ratio is commonly observed in obese individuals, and its reduction has been positively correlated with fat loss (Crovesy et al., 2020). However, the results among included studies were inconsistent, making it difficult to determine whether exercise directly reduces the F/B ratio. This observation aligns with previous findings, indicating that further research is needed to clarify the relationship between exercise and microbial composition. Finally, we observed that exercise may reduce the abundance of potentially pathogenic bacteria, such as *Bacteroides* and *Prevotella*, which could help mitigate gut dysbiosis in individuals with obesity and T2D. It is also important to note that, although this study has identified several potential patterns regarding specific microbial taxa and their associated metabolites, the conclusions should be interpreted with caution given the heterogeneity among the included studies in terms of intervention protocols, participant characteristics, and sequencing methodologies. Future research should employ larger sample sizes and high-quality randomized controlled trials, integrating multi-omics approaches (e.g., metagenomics, metabolomics, and longitudinal follow-up) to elucidate the mechanisms by which exercise modulates gut microbiota to improve obesity and T2D, and to explore the feasibility of personalized exercise prescriptions.

Finally, diet and medication are also key determinants of gut microbiota composition (David et al., 2014), and the effects of exercise on the gut microbiome may be intertwined with dietary intake and medication background. Evidence suggests that short-term macronutrient modifications can rapidly reshape community structure and alter microbial transcriptional profiles within just a few days, with effect sizes sufficient to overshadow interindividual variation (David et al., 2014). Long-term dietary patterns (e.g., the Mediterranean diet) and greater availability of fermentable substrates have been linked to butyrate-enriched communities and more favorable metabolomic profiles; these factors may amplify or mask the exercise-derived signals themselves (De Filippis et al., 2016). Regarding medications, metabolic drugs (particularly metformin) have been shown to significantly alter gut microbiota composition in individuals with T2D and to influence certain therapeutic outcomes (Wu et al., 2017). Antibiotic exposure and probiotic interventions can also induce substantial microbial shifts (Cammarota et al., 2014). In this study, we extracted and reported available information on dietary and medication control wherever possible and continuously acknowledged potential confounding in the interpretation. Nonetheless, in the absence of standardized dietary and medication management, these factors remain potential sources of residual confounding. Therefore, future studies adopting standardized and verifiable dietary management protocols, stable medication strategies, and exposure documentation during the intervention period will be essential to contextualize and accurately interpret the effects of exercise interventions.

## 5 Limitations

This study has several limitations. First, although we attempted to reduce heterogeneity through strict inclusion/exclusion criteria and subgroup analyses, variability in exercise prescriptions remained a major constraint. The included studies differed markedly in exercise type (aerobic, resistance, or combined), intensity settings, frequency, and intervention duration. Some studies only reported broad prescriptions without standardized documentation of load monitoring, adherence, or individualized adjustments, thereby increasing uncertainty in cross-study comparisons and interpretation of results. Second, inconsistencies in microbiome assessment methods also hindered evidence integration: variations across studies in sampling strategies, sequencing platforms and depth, and statistical adjustments led to differences in taxonomic resolution and functional inference, limiting direct causal interpretations along the “exercise–microbiome” pathway.

At the study design level, the included literature encompassed both randomized controlled trials and non-randomized studies, making methodological heterogeneity and potential bias unavoidable. We distinguished study designs in the study characteristics and risk-of-bias assessments and downgraded relevant outcomes in the GRADE evidence grading; however, these differences may still affect the external validity and causal interpretation of the conclusions. Moreover, most trials did not include healthy control groups, making it difficult to determine whether the observed microbiological changes reflect restorative shifts toward a healthy state or compensatory adaptations under disease conditions. Finally, some α-diversity outcomes (e.g., Simpson index and Observed OTUs) were reported in only a small number of studies, reducing statistical power and increasing uncertainty. Although we performed sensitivity analyses and graded presentation to enhance robustness and transparency, these results should still be interpreted with caution.

In summary, future research should aim for a higher degree of standardization and contextualization in two key areas. First, exercise interventions should provide reproducible prescriptions and objective adherence monitoring (specifying type, intensity [e.g.,%VO_2_max/%HRR/RPE, 1RM/sets/training volume], frequency, and duration, supplemented by verification via wearable devices and training logs). Second, microbiome assessments should adopt standardized sampling and quality-control protocols, apply uniform statistical approaches (e.g., compositional data analysis and FDR correction), and align taxonomic databases/versions to improve cross-study comparability and the feasibility of subsequent meta-analyses. Furthermore, it is recommended that future studies systematically include healthy control groups, expand sample sizes, and prioritize high-quality multicenter randomized controlled trials. Comprehensive reporting of α/β diversity, representative taxa, and key metabolites will enhance the reproducibility of evidence and its translational value in clinical practice.

## 6 Conclusion

In summary, exercise improves gut microbiota composition in individuals with obesity and T2D, primarily by increasing species richness, as reflected in the Shannon index, and by enhancing the abundance of rare microbial species, as indicated by the Chao1 index. Additionally, exercise may promote the proliferation of beneficial bacterial taxa. Compared to aerobic exercise alone, combined exercise interventions proved to be more effective, and younger individuals appeared to derive greater benefits from exercise interventions.

## Data Availability

The original contributions presented in this study are included in this article/Supplementary material, further inquiries can be directed to the corresponding author.

## Footnotes

1
https://automeris.io/WebPlotDigitizer

2
https://www.math.hkbu.edu.hk/~tongt/papers/median2mean.html
