# Supplementary material for: Exercise-induced modulation of gut microbiota in individuals with obesity and type 2 diabetes: a systematic review and meta-analysis
Source: Front Microbiol. 2025 Sep 24;16:1671975. doi: 10.3389/fmicb.2025.1671975 (PMC12504204; doi:10.3389/fmicb.2025.1671975)
Supplement: Supplementary file 1 [file Supplementary_file_1.docx]

*Supplementary materials*

| **Section and Topic** | **Item #** | **Checklist item** | **Location where item is reported** |
| --- | --- | --- | --- |
| **TITLE** | | |  |
| Title | 1 | Identify the report as a systematic review. | Done |
| **ABSTRACT** | | |  |
| Abstract | 2 | See the PRISMA 2020 for Abstracts checklist. | In abstract |
| **INTRODUCTION** | | |  |
| Rationale | 3 | Describe the rationale for the review in the context of existing knowledge. | In introduction |
| Objectives | 4 | Provide an explicit statement of the objective(s) or question(s) the review addresses. | In introduction |
| **METHODS** | | |  |
| Eligibility criteria | 5 | Specify the inclusion and exclusion criteria for the review and how studies were grouped for the syntheses. | methods (2.5) |
| Information sources | 6 | Specify all databases, registers, websites, organisations, reference lists and other sources searched or consulted to identify studies. Specify the date when each source was last searched or consulted. | methods (2.2) |
| Search strategy | 7 | Present the full search strategies for all databases, registers and websites, including any filters and limits used. | Table S1 |
| Selection process | 8 | Specify the methods used to decide whether a study met the inclusion criteria of the review, including how many reviewers screened each record and each report retrieved, whether they worked independently, and if applicable, details of automation tools used in the process. | methods (2.4) |
| Data collection process | 9 | Specify the methods used to collect data from reports, including how many reviewers collected data from each report, whether they worked independently, any processes for obtaining or confirming data from study investigators, and if applicable, details of automation tools used in the process. | methods (2.6) |
| Data items | 10a | List and define all outcomes for which data were sought. Specify whether all results that were compatible with each outcome domain in each study were sought (e.g. for all measures, time points, analyses), and if not, the methods used to decide which results to collect. | Table S3 |
|  | 10b | List and define all other variables for which data were sought (e.g. participant and intervention characteristics, funding sources). Describe any assumptions made about any missing or unclear information. | Table S3 |
| Study risk of bias assessment | 11 | Specify the methods used to assess risk of bias in the included studies, including details of the tool(s) used, how many reviewers assessed each study and whether they worked independently, and if applicable, details of automation tools used in the process. | methods (2.8) |
| Effect measures | 12 | Specify for each outcome the effect measure(s) (e.g. risk ratio, mean difference) used in the synthesis or presentation of results. | methods (2.9) |
| Synthesis methods | 13a | Describe the processes used to decide which studies were eligible for each synthesis (e.g. tabulating the study intervention characteristics and comparing against the planned groups for each synthesis (item #5)). | methods (2.9) |
|  | 13b | Describe any methods required to prepare the data for presentation or synthesis, such as handling of missing summary statistics, or data conversions. | methods (2.7) |
|  | 13c | Describe any methods used to tabulate or visually display results of individual studies and syntheses. | methods (2.9) |
|  | 13d | Describe any methods used to synthesize results and provide a rationale for the choice(s). If meta-analysis was performed, describe the model(s), method(s) to identify the presence and extent of statistical heterogeneity, and software package(s) used. | methods (2.9) |
|  | 13e | Describe any methods used to explore possible causes of heterogeneity among study results (e.g. subgroup analysis, meta-regression). | methods (2.9) |
|  | 13f | Describe any sensitivity analyses conducted to assess robustness of the synthesized results. | methods (2.9) |
| Reporting bias assessment | 14 | Describe any methods used to assess risk of bias due to missing results in a synthesis (arising from reporting biases). | methods (2.8) |
| Certainty assessment | 15 | Describe any methods used to assess certainty (or confidence) in the body of evidence for an outcome. | methods (2.10) |
| **RESULTS** | | |  |
| Study selection | 16a | Describe the results of the search and selection process, from the number of records identified in the search to the number of studies included in the review, ideally using a flow diagram. | Results (3.1) |
|  | 16b | Cite studies that might appear to meet the inclusion criteria, but which were excluded, and explain why they were excluded. | Figure 1 |
| Study characteristics | 17 | Cite each included study and present its characteristics. | Results (3.2) |
| Risk of bias in studies | 18 | Present assessments of risk of bias for each included study. | Results (3.5) |
| Results of individual studies | 19 | For all outcomes, present, for each study: (a) summary statistics for each group (where appropriate) and (b) an effect estimate and its precision (e.g. confidence/credible interval), ideally using structured tables or plots. | Results (3.3) |
| Results of syntheses | 20a | For each synthesis, briefly summarise the characteristics and risk of bias among contributing studies. | Results (3.5) |
|  | 20b | Present results of all statistical syntheses conducted. If meta-analysis was done, present for each the summary estimate and its precision (e.g. confidence/credible interval) and measures of statistical heterogeneity. If comparing groups, describe the direction of the effect. | Results (3.3) |
|  | 20c | Present results of all investigations of possible causes of heterogeneity among study results. | Results (3.5) |
|  | 20d | Present results of all sensitivity analyses conducted to assess the robustness of the synthesized results. | Results (3.5.3) |
| Reporting biases | 21 | Present assessments of risk of bias due to missing results (arising from reporting biases) for each synthesis assessed. | Results (3.5.2) |
| Certainty of evidence | 22 | Present assessments of certainty (or confidence) in the body of evidence for each outcome assessed. | Results (3.5.5) |
| **DISCUSSION** | | |  |
| Discussion | 23a | Provide a general interpretation of the results in the context of other evidence. | In Discussion |
|  | 23b | Discuss any limitations of the evidence included in the review. | In Discussion |
|  | 23c | Discuss any limitations of the review processes used. | In Discussion |
|  | 23d | Discuss implications of the results for practice, policy, and future research. | In Discussion |
| **OTHER INFORMATION** | | |  |
| Registration and protocol | 24a | Provide registration information for the review, including register name and registration number, or state that the review was not registered. | Methods (2.1) |
|  | 24b | Indicate where the review protocol can be accessed, or state that a protocol was not prepared. | Methods (2.1) |
|  | 24c | Describe and explain any amendments to information provided at registration or in the protocol. | Methods (2.1) |
| Support | 25 | Describe sources of financial or non-financial support for the review, and the role of the funders or sponsors in the review. | Reported |
| Competing interests | 26 | Declare any competing interests of review authors. | Reported |
| Availability of data, code and other materials | 27 | Report which of the following are publicly available and where they can be found: template data collection forms; data extracted from included studies; data used for all analyses; analytic code; any other materials used in the review. | Reported |

**2. supplementary materials Table S2. search strategy in PubMed, Embase, Cochrane Library and Web of Science** **databases. All Databases Search deadline:2025\2\17**

**PubMed**

| step | Search | **Hits** |
| --- | --- | --- |
| #1 | (((((((((((((((((((("Diabetes Mellitus, Type 2"[Mesh]) OR ("Diabetes Mellitus"[Mesh])) OR (Diabetes)) OR (Diabetic)) OR (MODY[Title/Abstract])) OR (NIDDM[Title/Abstract])) OR (Diabetes Mellitus, Adult-Onset[Title/Abstract])) OR (Ketosis-Resistant Diabetes Mellitus[Title/Abstract])) OR (Diabetes Mellitus, Stable[Title/Abstract])) OR (Diabetes Mellitus, Type II[Title/Abstract])) OR (Diabetes Mellitus, Noninsulin Dependent[Title/Abstract])) OR (Diabetes Mellitus, Maturity-Onset[Title/Abstract])) OR (Maturity-Onset Diabetes Mellitus[Title/Abstract])) OR (Diabetes Mellitus, Slow-Onset[Title/Abstract])) OR (Type 2 Diabetes Mellitus[Title/Abstract])) OR (Noninsulin-Dependent Diabetes Mellitus[Title/Abstract])) OR (Maturity-Onset Diabetes[Title/Abstract])) OR (Diabetes, Maturity-Onset[Title/Abstract])) OR (Type 2 Diabetes[Title/Abstract])) OR (Diabetes, Type 2[Title/Abstract])) OR (Diabetes Mellitus, Noninsulin-Dependent[Title/Abstract]) | **1017466** |
| #2 | (((((((((("Obesity"[Mesh]) OR "Overweight"[Mesh]) OR "Pediatric Obesity"[Mesh]) OR (Obesity, Abdominal[Title/Abstract])) OR (Child Obesity[Title/Abstract])) OR (Adolescent Obesity[Title/Abstract])) OR (Abdominal Obesity[Title/Abstract])) OR (Central Obesity[Title/Abstract])) OR (Visceral Obesity[Title/Abstract])) OR (Obesity)) OR (Overweight) | **512433** |
| #3 | (("Hypertension"[Mesh]) OR (High Blood Pressure)) OR (Hypertension) | **787634** |
| #4 | ((((((((((("Metabolic Syndrome"[Mesh]) OR (Metabolic Syndromes)) OR (Metabolic Cardiovascular Syndrome)) OR (Cardiometabolic Syndrome)) OR (Insulin Resistance Syndrome X)) OR (Dyslipidemias)) OR (Coronary Disease)) OR (Stroke)) OR (Atherosclerosis)) OR (Atrial Fibrillation)) OR (Heart Failure)) OR (Cardiovascular Diseases) | **3389363** |
| #5 | #1OR#2OR#3OR#4 | **4599753** |
| #6 | (((((((((((((((((((("Exercise"[Mesh]) OR "Sports"[Mesh]) OR "Resistance Training"[Mesh]) OR (Aerobic Exercise)) OR (resistance training)) OR (Physical Exercise)) OR (Isometric Exercise)) OR (plyometric exercise)) OR (strength training)) OR (Acute Exercise)) OR (Exercise Training)) OR (running)) OR (cycling)) OR (swim)) OR (fitness)) OR (hiking)) OR (jogging)) OR (walking)) OR (Exercise)) OR (Physical Activity)) OR (Sports) | **2114683** |
| #7 | (((((((((((((((((("Gastrointestinal Microbiome"[Mesh]) OR (Gut Microbiome)) OR (Gut Microflora)) OR (Gut Flora)) OR (Gastrointestinal Microbiome)) OR (microbiota)) OR (prebiotics)) OR (probiotics)) OR (Gastrointestinal Microbial Community)) OR (Gastrointestinal Microflora)) OR (Gastrointestinal Flora)) OR (Gastrointestinal Microbiota)) OR (Gut Microbiota)) OR (Intestinal Microbiome)) OR (Intestinal Flora)) OR (Intestinal Microbiota)) OR (Intestinal Microflora)) OR (Enteric Bacteria)) OR (Gastric Microbiome) | **220001** |
| #8 | #5AND#6AND#7 | **2102** |
| Limits: human, English,RCT | | |

**Embase（Medline）**

| step | Search | **Hits** |
| --- | --- | --- |
| #1 | non AND ('insulin'/exp OR insulin) AND dependent AND ('diabetes'/exp OR diabetes) AND mellitus OR (('diabetes'/exp OR diabetes) AND mellitus) OR 'adult onset diabetes mellitus':ti,ab,kw OR 'diabetes mellitus type 2':ti,ab,kw OR 'diabetes type 2':ti,ab,kw OR 'diabetes type ii':ti,ab,kw OR 'insulin independent diabetes':ti,ab,kw OR 'ketosis resistant diabetes mellitus':ti,ab,kw OR 'maturity onset diabetes':ti,ab,kw OR niddm:ti,ab,kw OR t2dm:ti,ab,kw OR tiidm:ti,ab,kw OR 'type 2 diabetes':ti,ab,kw OR 'type ii diabetes':ti,ab,kw OR diabetes:ti,ab,kw OR diabetic:ti,ab,kw OR diabets:ti,ab,kw OR 'unspecified diabetes mellitus':ti,ab,kw OR 'non insulin dependent diabetes mellitus'/exp OR 'non insulin dependent diabetes mellitus' OR 'diabetes mellitus'/exp OR 'diabetes mellitus' | **1606500** |
| #2 | 'obesity'/exp OR 'obesity' OR 'adipose tissue hyperplasia':ti,ab,kw OR adipositas:ti,ab,kw OR adiposity:ti,ab,kw OR 'alimentary obesity':ti,ab,kw OR 'body weight, excess':ti,ab,kw OR corpulency:ti,ab,kw OR 'fat overload syndrome':ti,ab,kw OR 'nutritional obesity':ti,ab,kw OR obesitas:ti,ab,kw OR overweight:ti,ab,kw OR obesity:ti,ab,kw | **883980** |
| #3 | 'hypertension'/exp OR hypertension OR 'high blood pressure':ti,ab,kw OR 'acute hypertension':ti,ab,kw OR 'arterial hypertension':ti,ab,kw OR 'cardiovascular hypertension':ti,ab,kw OR 'controlled hypertension':ti,ab,kw OR 'endocrine hypertension':ti,ab,kw OR 'high renin hypertension':ti,ab,kw OR (htn:ti,ab,kw AND hypertension:ti,ab,kw) OR 'hypertensive disease':ti,ab,kw OR 'hypertensive effect':ti,ab,kw OR 'hypertensive reaction':ti,ab,kw OR 'hypertensive response':ti,ab,kw OR 'neurogenic hypertension':ti,ab,kw OR 'preexistent hypertension':ti,ab,kw OR 'salt high blood pressure':ti,ab,kw OR 'salt hypertension':ti,ab,kw OR 'secondary hypertension':ti,ab,kw OR 'systemic hypertension':ti,ab,kw | **1450780** |
| #4 | 'metabolic syndrome x'/exp OR 'dyslipidemia'/exp OR 'coronary artery disease'/exp OR 'cerebrovascular accident'/exp OR 'atherosclerosis'/exp OR 'atrial fibrillation'/exp OR 'heart failure'/exp OR metabolic AND syndrome AND x OR (insulin AND resistance AND syndrome) OR metsyn OR (reaven AND syndrome) OR dyslipidemia OR (coronary AND artery AND disease) OR (coronary AND disease) OR (cerebrovascular AND accident) OR (acute AND stroke) OR stroke OR atherosclerosis OR (atrial AND fibrillation) OR (heart AND failure) OR (cardiac AND failure) | **2630325** |
| #5 | #1OR#2OR#3OR#4 | **4995447** |
| #6 | 'exercise'/exp OR 'physical activity'/exp OR 'sport'/exp OR 'resistance training'/exp OR 'resistance training' OR (('resistance'/exp OR resistance) AND ('training'/exp OR training)) OR 'physical exercise':ti,ab,kw OR 'isometric exercise':ti,ab,kw OR plyometrics:ti,ab,kw OR 'strength training':ti,ab,kw OR 'acute exercise':ti,ab,kw OR 'exercise training':ti,ab,kw OR running:ti,ab,kw OR cycling:ti,ab,kw OR swim:ti,ab,kw OR fitness:ti,ab,kw OR hiking:ti,ab,kw OR jogging:ti,ab,kw OR walking:ti,ab,kw OR exercise OR (physical AND activity) OR sport | **1856850** |
| #7 | 'intestine flora'/exp OR 'intestine flora' OR (('intestine'/exp OR intestine) AND ('flora'/exp OR flora)) OR (bowel AND flora) OR (bowel AND microbiota) OR (enteric AND flora) OR (enteric AND microbiota) OR (gastro AND intestinal AND flora) OR (gastrointestinal AND flora) OR (gastrointestinal AND microbiome) OR (gastrointestinal AND microbiota) OR (gastrointestinal AND tract AND flora) OR (gut AND bacteria) OR (gut AND microbiota) OR (intestinal AND bacteria) OR (intestinal AND bacterial AND flora) OR (intestinal AND bacterium) OR (intestinal AND flora) OR (intestinal AND microbe) OR (intestinal AND microbes) OR (intestinal AND microbiota) OR (intestinal AND microflora) OR (intestinal AND microorganism) OR (intestine AND bacteria) OR (intestine AND microflora) | **260136** |
| #8 | #5AND#6AND#7 | **2943** |

**Web of Science**

| step | Search | **Hits** |
| --- | --- | --- |
| #1 | (((((((((((((((((((((ALL=(Diabetes Mellitus)) OR ALL=(Diabetes Mellitus, Type 2)) OR ALL=(Diabetic)) OR TS=(Diabet)) OR TS=(MODY)) OR TS=(NIDDM)) OR TS=(Type 2 Diabetes)) OR ALL=(Obesity)) OR ALL=(Pediatric Obesity)) OR ALL=(Overweight)) OR ALL=(Metabolic Syndrome)) OR ALL=(Cardiometabolic Syndrome)) OR ALL=(Hypertension)) OR ALL=(High Blood Pressure)) OR ALL=(Dyslipidemias)) OR ALL=(Coronary Disease)) OR ALL=(Stroke)) OR ALL=(Atherosclerosis)) OR ALL=(Atrial Fibrillation)) OR ALL=(Heart Failure)) OR ALL=(Cardiovascular Diseases)) OR ALL=(Cardiometabolic Diseases) | **3131849** |
| #2 | (((((((((((((((((ALL=(Exercise)) OR ALL=(Physical Activity)) OR ALL=(Sports)) OR TS=(Aerobic Exercise)) OR TS=(resistance training)) OR TS=(Physical Exercise)) OR TS=(Isometric Exercise)) OR TS=(plyometrics)) OR TS=(strength training)) OR TS=(Acute Exercise)) OR TS=(Exercise Training)) OR TS=(running)) OR TS=(cycling )) OR TS=(swim)) OR TS=(fitness )) OR TS=(hiking)) OR TS=(jogging)) OR TS=(walking) | **4797808** |
| #3 | ((((((((((ALL=(Gastrointestinal Microbiome)) OR ALL=(Gut Microbiome)) OR ALL=(Gut Microflora)) OR ALL=(Gut Flora)) OR ALL=(Gut Microbiota)) OR TS=(microbiota)) OR TS=(prebiotics)) OR TS=(probiotics)) OR TS=(Intestinal Microbiome)) OR TS=(Enteric Bacteria)) OR TS=(Gastric Microbiome) | **214870** |
| #4 | #1 AND #2 AND #3 | **3164** |
|  |  |  |

**Cochrane Library**

| Search Name:  Date Run: 21/11/2024 08:24:24  Comment:  ID Search Hits  #1 (Diabetes Mellitus, Maturity Onset OR Ketosis-Resistant Diabetes Mellitus OR Diabetes, Maturity-Onset OR Type 2 Diabetes Mellitus OR Diabetes Mellitus, Slow Onset OR Diabetes Mellitus, Adult-Onset OR NIDDM OR Type 2 Diabetes OR Diabetes Mellitus, Non-Insulin-Dependent OR Diabetes Mellitus, Non Insulin Dependent OR Diabetes Mellitus, Maturity-Onset OR Adult-Onset Diabetes Mellitus OR Diabetes Mellitus, Noninsulin Dependent OR Diabetes Mellitus, Stable OR Non-Insulin-Dependent Diabetes Mellitus OR Maturity-Onset Diabetes Mellitus OR Diabetes Mellitus, Slow-Onset OR MODY OR Slow-Onset Diabetes Mellitus OR Maturity Onset Diabetes OR Maturity-Onset Diabetes OR Maturity Onset Diabetes Mellitus OR Stable Diabetes Mellitus OR Diabetes, Type 2 OR Noninsulin-Dependent Diabetes Mellitus OR Diabetes Mellitus, Ketosis Resistant OR Noninsulin Dependent Diabetes Mellitus OR Diabetes Mellitus, Noninsulin-Dependent OR Diabetes Mellitus, Adult Onset OR Diabetes Mellitus, Ketosis-Resistant OR Diabetes Mellitus, Type II):ti,ab,kw (Word variations have been searched) **70707**  #2 MeSH descriptor: [Diabetes Mellitus] explode all trees **46942**  #3 MeSH descriptor: [Diabetes Mellitus, Type 2] explode all trees **26834**  #4 #1or#2or#3 **84054**  #5 MeSH descriptor: [Obesity] explode all trees **21908**  #6 MeSH descriptor: [Overweight] explode all trees **25675**  #7 MeSH descriptor: [Pediatric Obesity] explode all trees **2261**  #8 #4or#5or#6or#7 105618  #9 MeSH descriptor: [Metabolic Syndrome] explode all trees **3640**  #10 MeSH descriptor: [Hypertension] explode all trees **25637**  #11 (Blood Pressure, High or High Blood Pressure or Blood Pressures, High or High Blood Pressures):ti,ab,kw (Word variations have been searched) **33503**  #12 (Dyslipidemias or Coronary Disease or Stroke or Atherosclerosis or Atrial Fibrillation or Heart Failure or Cardiac Failure or Cardiovascular Diseases):ti,ab,kw (Word variations have been searched) **205224**  #13 #8or#9or#10or#11or#12 **325625**  #14 MeSH descriptor: [Exercise] explode all trees **39777**  #15 (Isometric Exercise OR Exercise, Isometric OR Exercises, Isometric OR Isometric Exercises OR Exercise Trainings OR Training, Exercise OR Exercise Training OR Trainings, Exercise OR Physical Exercises OR Exercises, Physical OR Physical Activity OR Activity, Physical OR Exercise, Physical OR Exercises OR Activities, Physical OR Physical Exercise OR Physical Activities OR Exercise, Aerobic OR Aerobic Exercise OR Exercises, Aerobic OR Aerobic Exercises OR Acute Exercises OR Exercise, Acute OR Exercises, Acute OR Acute Exercise OR running OR cycling OR swim OR fitness OR hiking OR jogging OR walking):ti,ab,kw (Word variations have been searched) **318550**  #16 #14 OR #15 **319426**  #17 MeSH descriptor: [Gastrointestinal Microbiome] explode all trees **1832**  #18 (Enteric Bacteria OR Bacteria, Enteric OR Gastric Microbiome OR Gastric Microbiomes OR Microbiome, Gastric OR Intestinal Microflora OR Intestinal Microbiota OR Intestinal Microbiome OR Intestinal Microbiotas OR Intestinal Flora OR Intestinal Microbiomes OR Microbiota, Intestinal OR Flora, Intestinal OR Microflora, Intestinal OR Microbiome, Intestinal OR Gastrointestinal Microbial Community OR Gut Flora OR Gut Microbiotas OR Gut Microbiomes OR Gut Microbiome OR Microbiota, Gastrointestinal OR Microflora, Gastrointestinal OR Gastrointestinal Microbiomes OR Flora, Gut OR Microflora, Gut OR Gut Microbiota OR Flora, Gastrointestinal OR Microbiota, Gut OR Microbiome, Gut OR Microbial Community, Gastrointestinal OR Gastrointestinal Flora OR Microbiome, Gastrointestinal OR Gastrointestinal Microflora OR Gut Microflora OR Gastrointestinal Microbiotas OR Gastrointestinal Microbiota OR Gastrointestinal Microbial Communities):ti,ab,kw (Word variations have been searched) **12112**  #19 #17 OR #18 **12112**  #20 #13AND#16AND#19 **446** |
| --- |

| **randomized controlled trial** |
| --- |
| **PubMed**  (randomized controlled trial[pt] OR controlled clinical trial[pt] OR randomized[tiab] OR randomised[tiab] OR placebo[tiab] OR "clinical trials as topic"[MeSH Terms] OR randomly[tiab] OR trial[ti]) AND (humans[mh]) |
| **Embase**  (randomized controlled trial'/exp OR 'controlled clinical trial'/exp OR randomized:ti,ab OR randomised:ti,ab OR placebo:ti,ab OR 'clinical trial'/exp OR randomly:ti,ab OR trial:ti) AND ('human'/exp) |
| **Web of Science**  TS=("randomized controlled trial" OR "randomised controlled trial" OR "RCT" OR "controlled clinical trial" OR randomized OR randomised OR placebo OR trial) AND TS=("human") |
| **Cochrane Library**  [MeSH descriptor: "Randomized Controlled Trials as Topic" explode all trees] OR ("randomized controlled trial" OR "randomised controlled trial" OR "RCT" OR "controlled clinical trial" OR randomized OR randomised OR placebo OR trial):ti,ab,kw AND [MeSH descriptor: "Humans" explode all trees] |

| **3. supplementary materials** **Table S3. Study characteristics** | | | | | | | |
| --- | --- | --- | --- | --- | --- | --- | --- |
| Study characteristics | | | | | | | |
| Author | Year | Sample Size | Intervention Type | Region | Sex | Condition | Age |
| John M. A. Cullen | 2024 | 16——16 | Resistance training program lasting 6 weeks, with 3 sessions per week | USA | Male & Female | Obesity | 25-35 |
| DUPUIT, MARINE | 2022 | 8——9 | HIIT + RT group (3 sessions per week for 12 weeks) | China | Female | Obesity | 50-65 |
| Gabriela Batitucci | 2023 | 20（11）——20（10） | HIIT training for 8 weeks (intervention group) | Brazil | Female | Obesity | 18-40 |
| Zongyu Lin | 2023 | 28——26 | 12-week RS group: rope skipping 3 times/week, 2,000 skips per session (100 skips/set, 20s rest). | China | Male & Female | Obesity | 23 |
| Shaodong Wei | 2021 | 49——19 | Participants performed aerobic exercise five to six times per week, with two to three sessions combined with resistance training. | Denmark | Male & Female | T2D, Obesity | 54+ |
| Yao Wang | 2024 | 20——19 | High-intensity interval training (HIIT), 3 sessions per week for 12 weeks | China | Male | T2D, Obesity | 20+ |
| Runtan Cheng | 2022 | 22——16 | Supervised progressive aerobic exercise (e.g., Nordic walking + stretching), 2–3 sessions/week, 30–60 min/session, 60–75% VO₂max (estimated) | China | Male & Female | T2D | 50-65 |
| Fei Zhong | 2022 | 8——6 | 4 sessions/week, 9:30–10:30 a.m.; 60 min/session: warm-up (10 min), aerobic (20 min), resistance (25 min), cool-down (5 min). | China | Female | T2D | 60 - 75 |
| Ky Young Cho | 2021 | 17——19 | Intensive lifestyle intervention (daily stretching, cycling after school/weekends, stair use instead of elevators when safe) | South Korea | Male | Obesity | Children |
| Timo Kern | 2020 | 24——14 | 6 months, 5 training days/week: CON (habitual lifestyle), BIKE (cycling commute), MOD (50% VO₂ peak reserve), or VIG (70% VO₂ peak reserve) | Denmark | Male & Female | Obesity | 20-45 |
| ALLEN，JACOB M. | 2018 | 14——18 | 6-week supervised aerobic training, 3 sessions/week, 30–60 min/session, 60%–75% HRR (cycle ergometer or treadmill). | USA | Male & Female | Obesity | 18——40 |
| L. Torquati | 2022 | 5——7 | C-HIIT: 3 sessions/week (26 min/session, 78 min/week); 3-min warm-up at 50–60% HRpeak + 4-min high-intensity intervals at 85–95% HRpeak (non-continuous). | Australia | Male & Female | T2D | 55-70 |
| Owen Cronin | 2018 | 12——11 | EP group: aerobic + resistance training, 3 sessions/week for 8 weeks | Ireland | Male & Female | Obesity | 18-40 |
| Martha Guevara‐Cruz | 2019 | 18——17 | Enhanced physical activity: step count monitored by pedometer; increased by 10% (first 15 days), 25% (next month), and 50% (second month) compared to baseline. | Mexico | Male & Female | Metabolic Syndrome | 20-65 |
|  |  | 13——10 |  |  |  | Obesity |  |
| Beals, JW | 2023 | 8——8 | 6 sessions/week (1 hour each); 4 supervised by exercise physiologist, 2 home-based. | USA | Male & Female | T2D, Obesity | 43+ |
| Libuša Nechalová | 2024 | 12——11 | During the 12-week intervention period, participants attended two supervised exercise sessions per week. | Slovakia | Male & Female | Obesity | 47.08 ±10.48 |
| Martin S. Lietzén | 2024 | （12）11——10（12） | 27 weeks; 2 endurance, 1 resistance, and 1 HIIT session per week | Finland | Male & Female | Obesity | Average45 |
| Jesús  F García-Gavilán | 2024 | 200——200 | Brisk walking ≥45 min/day or equivalent + targeted strength, balance, and flexibility exercises; goal: ≥150 min/week of MVPA | Spain | Male & Female | T2D, Obesity | 64+ |
| Jukka E. Hintikka | 2023 | 17——17 | 3 sessions/week; Weeks 1–2: 40 min low-intensity cycling; Weeks 3–4: 50 min (alternate sessions with 3×10 min moderate-intensity intervals); Weeks 5–6: 60 min (with 4×10 min moderate-intensity intervals); remaining time low intensity. | Finland | Female | Obesity | 36 |

**4. supplementary materials Table S4. Beta Diversity**

| Methodology and findings of the included studies assessing beta diversity for  the patient vs. control group comparison. | | | | | |
| --- | --- | --- | --- | --- | --- |
| **Study** | **Year** | **Metric** | **Analysis** | **Finding** | **Disorder** |
| John | 2024 | Unweighted Unifrac Weighted Unifrac Bray Curtis | PCoA | no sig difference no sig difference no sig difference | Obesity |
| DUPUIT | 2022 | Unweighted Unifrac Weighted Unifrac | PCoA | sig difference  sig difference | Obesity |
| Gabriela | 2023 | Bray Curtis | PCoA | no sig difference | Obesity |
| Lin | 2021 | Bray Curtis | PCoA | sig difference | Obesity |
| Shao | 2023 | Weighted Unifrac | PCoA | sig difference | T2D  Obesity |
| Wang | 2024 | Bray-Curtis | —— | sig difference | T2D  Obesity |
| Cheng | 2022 | Unweighted Unifrac Weighted Unifrac | PCoA | sig difference  sig difference | T2D |
| Fei | 2022 | —— | —— | —— | T2D |
| Cho | 2021 | Unweighted Unifrac Weighted Unifrac | PCoA | sig difference  sig difference | Obesity |
| Kern | 2020 | Weighted Unifrac Bray Curtis | PERMANOVA | sig difference  sig difference | Obesity |
| ALLEN | 2018 | Unweighted Unifrac Weighted Unifrac | PERMANOVA PCoA | no sig difference no sig difference | Obesity |
| Torquati | 2022 | Unweighted Unifrac Weighted Unifrac | PERMANOVA PCoA | sig difference  sig difference | T2D |
| Cronin | 2018 | Bray-Curtis | PCoA | sig difference | Obesity |
| Martha | 2019 | Unweighted Unifrac Weighted Unifrac | PCoA | sig difference  sig difference | T2D  Obesity |
| Beals | 2023 | Weighted Unifrac | PERMANOVA | sig difference | T2D  Obesity |
| Wu | 2023 | Bray-Curtis | PERMANOVA PCoA | sig difference | T2D  Obesity |
| Hirokazu Taniguchi | 2018 | Unweighted Unifrac Weighted Unifrac Bray Curtis | PCoA | no sig difference no sig difference no sig difference | T2D  Obesity |
| Jukka E. Hintikka | 2023 | —— | —— | —— | Obesity |
| Libuša Nechalová | 2018 | —— | —— | —— | Obesity |

PCoA :principal coordinates analysis; PERMANOVA = permutational analysis of variance; PERMANOVA: permutational analysis of varian

**5. supplementary materials FigureS1. Subgroup Analysis of Intervention Duration**

| **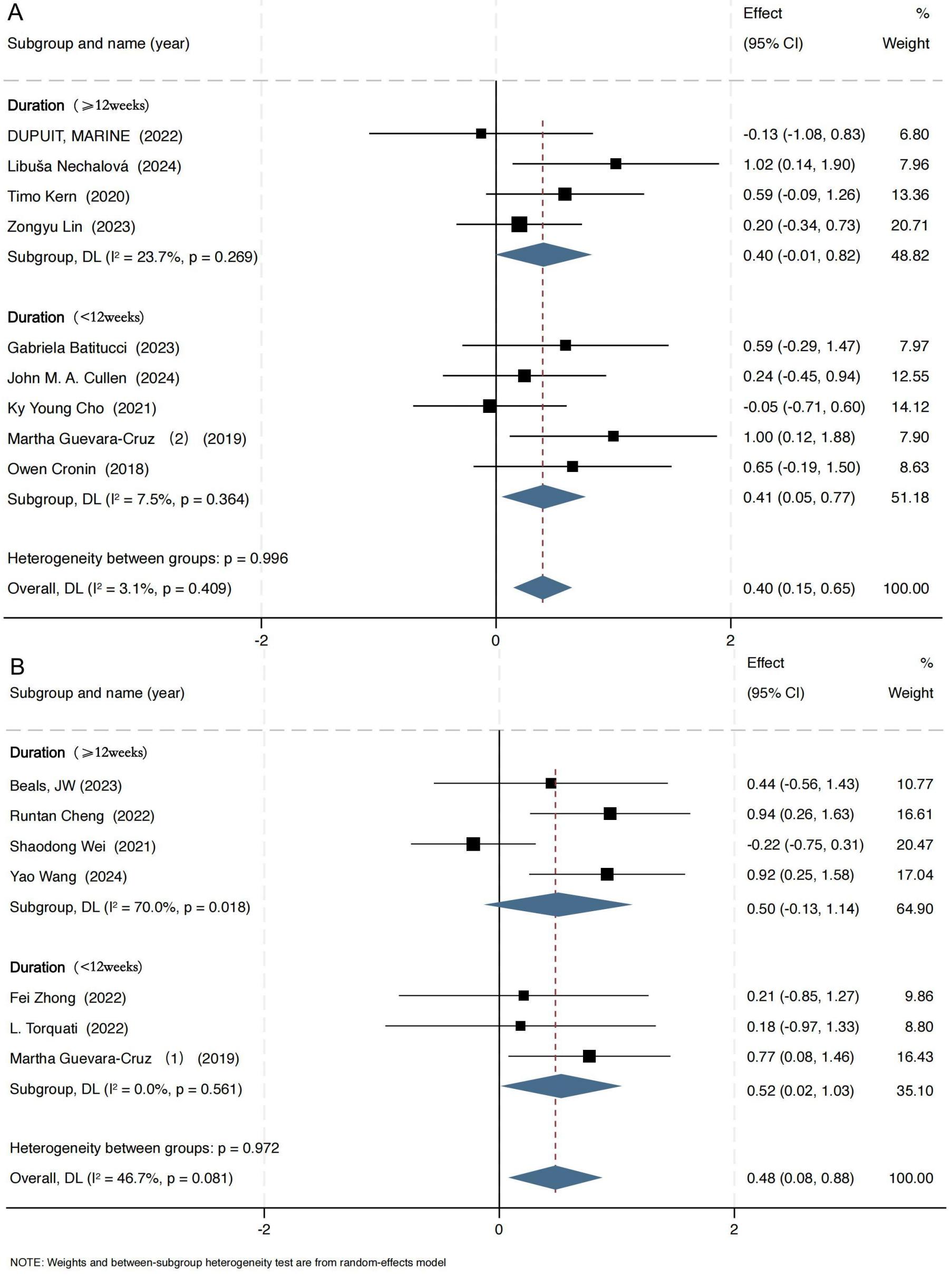** |
| --- |
| Obesity(S1A), Diabetes(S1B) |

**6. supplementary materials Table S5. Meta-regression**

| Variable | | *β* | 95% CI | z | *P* |
| --- | --- | --- | --- | --- | --- |
| Country/Region | Asia (vs. Americas) | -0.151 | -0.741~ 0.439 | -0.50 | 0.615 |
|  | Europe (vs. Americas) | -0.273 | -0.878 ~ 0.332 | -0.88 | 0.376 |
|  | Oceania (vs. Americas) | -0.418 | -1.767 ~ 0.930 | -0.61 | 0.543 |
| Intervention duration | ≥12 weeks (vs. <12 weeks) | -0.0036 | -0.472 ~ 0.464 | -0.02 | 0.988 |
| Age | >50 years (vs. ≤50 years) | -0.3475 | -0.835 ~ 0.140 | -1.40 | 0.163 |
| Gender | Female-dominant (vs. Male-dominant) | 0.2707 | -0.1714 ~ 0.7128 | 1.20 | 0.230 |
| Exercise type | Aerobic vs. Combined | 0.1083 | -0.3595 ~ 0.5760 | 0.45 | 0.650 |

**7. supplementary materials Table S6. Summary of Gut Microbiota and Metabolite Changes After Exercise**

| Study | Upregulated genera | Downregulated genera | Other microbial changes | SCFAs | Other metabolite | Notes |
| --- | --- | --- | --- | --- | --- | --- |
| Beals  2023 | NR | NR | NR | NR | NR | —— |
| Ky Young Cho2021 | *Romboutsia, Ruminococcaceae UCG_013, Eubacterium coprostanoligenes group, and Parabacteroides* | *Bacteroides* and *Oscillibacter* (note: *Bacteroides* also decreased in the control group); | ↑*Firmicutes, Clostridia/Clostridiales;* ↓*Bacteroidetes, Bacteroidia/Bacteroidales, Bacteroidaceae, Bacteroides; Dorea, Blautia, Fusicatenibacter, and Clostridium sensu stricto 1* elevated in early phase with unclear later trends | NR | NR | NetShift network analysis; ALDEx2 differential abundance testing; SCFAs, LPS, and bile acids not reported |
| Owen Cronin2018 | *Prevotella copri* | NR | ↑ *Actinobacteria and Verrucomicrobia; Firmicutes* | NR | ↓ PAG and TMAO; ↑ glutamate | 619 unadjusted differential pathways; significant β-diversity shifts in the exercise group; Prevotella identified by PLS-DA |
| Jesús  F García-Gavilán2024 | None | *Eubacterium hallii；Dorea* | NR | NR | Metabolomics: ↑4,7,10,13,16-DPA and oleic acid; ↓3-MAA and adrenic acid，ceramide module, fattyacid/carnitine module | Significant shifts in metabolite networks correlated with HOMA-IR and LDL; SCFAs and LPS not reported |
| Jukka E. Hintikka2023 | *Akkermansia* | NR | *Verrucomicrobiota* | NR | ↑Fecal metabolites: glycerophosphocholine, proline betaine, histidine–proline, inositol; enriched in glycerophospholipid, taurine, and amino acid metabolism | Metabolic changes closely associated with increased Akkermansia abundance; SCFAs not directly measured |
| Libuša Nechalová2024 | *Akkermansia muciniphila Parabacteroides merdae Phocaeicola vulgatus* | *Butyrivibrio fibrisolvens,* Coprococcus comes, *Blautia spp. (B. hydrogenotrophica, B. massiliensis), Erysipelatoclostridium ramosum, Faecalibacterium* | ↑*Bacteroidetes,*  ↑*Verrucomicrobiota;*  ↓*Firmicutes,*  ↓*Firmicutes/Bacteroidetes ratio* | SCFA-producing bacteria significantly decreased. fecal SCFA levels showed no significant difference | No significant changes in fecal metabolites | P. vulgatus negatively correlated with body fat and visceral adiposity |
| L. Torquati2022 | *Bifidobacterium spp., Escherichia spp., Akkermansia municiphila, Lachnospira eligens, Enterococcus spp., Clostridium cluster* IV (*C. leptum, F. prausnitzii*), *Ruminococcus bromii* | NR | No significant changes | No significant changes | NR | Increased abundance of butyrate-producing bacteria in the exercise group |
| ALLEN，JACOB M.2018 | *Roseburia spp.- Lachnospira spp.- Faecalibacterium spp.- Clostridiales spp.- Lachnospiraceae unclassified- Clostridium spp.- Eubacterium spp.- Methanobrevibacter smithii* | Bacteroides spp.- Rikenella spp. | NR | Acetate, propionate, and butyrate significantly increased during training (P < 0.05); SCFA levels declined after exercise cessation | Increased expression of butyrate-related *gene BCoAT* and propionate-related gene mmdA during intervention | Changes in certain genera were consistent with the improvement in VO₂max |
| Martin S. Lietzén2024 | *Megamonas, Helicobacter, Limosilactobacillus, Lactobacillus, CHKCL001, Propionibacterium, Xanthomonas, Enorma, Staphylococcus* | NR | Mid-intervention: ↑*Firmicutes,* ↓*Bacteroidetes* and *Proteobacteria* (pFDR < 0.05);↑*Campilobacterota* | NR | NR | Phylum-level shifts observed only during mid-intervention and returned to baseline post-intervention; analyzed using linear mixed models with FDR correction |
| Runtan Cheng2022 | *Bacteroides* (ASV2077, 2440, 1989), *Ruminococcus* (ASV3942, 3307), *Alisma* (ASV1715), *Koellia* (ASV478), *Lachnoclostridium* (ASV5195, 5305)*，Ruminiclostridium* (ASV4538) | NR | ↑*Ruminococcaceae* (ASV5361), *Ruminococcaceae* UCG-XXX (ASV3307), *Erysipelotrichaceae* (ASV776) | No significant differences | NR | Six fecal SCFAs measured from supernatant using gas chromatography (GC) |
| John M. A. Cullen2024 | *Roseburia*↑ *R. faecis*↑  *Roseburia sp.*↑ *F. prausnitzii*↑ | *Holdemania*（ASV）↓ | ↑*Firmicutes* (phylum);  ↑*Lachnospiraceae*  ↑*Ruminococcaceae* (family) | SCFAs not directly measured | Microbial metabolism and cell motility pathways showed marginal significance (P < 0.05, q < 0.25) | *Roseburia* is a key SCFA-producing genus of interest |
| Martha Guevara‐Cruz2019 | *Akkermansia muciniphila*↑, *Faecalibacterium prausnitzii*↑, *Ruminococcus*↑, *Parabacteroides*↑, *Selenomonas*↑, *Bacteroides ovatus*↑, *Roseburia*↑ | *Prevotella*↓, *Bacteroides*↓ | *Bacteroidetes, Firmicutes,* and *Proteobacteria* accounted for approximately 97% of total relative abundance | SCFA levels were not directly reported, increased abundance of *F. prausnitzii* and *Roseburia* suggests a potential enhancement in SCFA metabolic function | NR | Intervention groups generally showed increased beneficial taxa, enhanced diversity, reduced Firmicutes/Bacteroidetes ratio, and a shift in gut microbiota composition toward a healthier profile |
| Timo Kern2020 | NR | NR | NR | NR | NR | NR |
| Zongyu Lin2023 | *Eubacterium_coprostanoligenes_group*  *Lachnospiraceae*  *Blautia* | *Lactobacillus Muribaculaceae* | No significant changes in *Bacteroidetes* and *Firmicutes* at the phylum level | NR | NR | 12-week exercise increased potential probiotics and suppressed Lactobacillus and Muribaculaceae, modulating gut microbiota composition |
| Yao Wang2024 | *Verticillium Sarocladium Ceratocystis, Chloridium, Iodophanus, Monosporascus, Beauveria*, *Bipolaris, Gliomastix,* and *Conocybe* increased | Only 2.13% of genera showed significant decreases; specific taxa not reported | NR | NR | NR | Increases in fungal genera such as *Verticillium* and *Sarocladium* were positively associated with improved insulin sensitivity (↓fasting insulin, ↓HOMA-IR) |
| Shaodong Wei2021 | *Bacteroides, Ruminococcaceae UCG-014, Alisma, Roseburia, Blautia, Escherichia, Faecalibacterium* | NR | NR | NR | NR | Fecal DNA extracted using an automated platform and taxonomic annotation performed based on the Silva database |
| DUPUIT, MARINE2022 | NR | NR | NR | NR | NR | —— |
| Fei Zhong2022 | *Roseburia, Ruminococcus torques group, Butyricicoccus, Parasutterella, Escherichia-Shigella, Holdemania* | *Lactobacillus, Muribaculaceae, Lachnospiraceae_NK4A136_group, Allobaculum, Bacteroides, Sutterella* | *Firmicutes/Bacteroidetes ratio significantly decreased;*  ↑*Betaproteobacteria* (class) and *Sutterellaceae* (family) | NR | NR | OTU clustering and annotation performed using standard pipelines (UPARSE, UCHIME, QIIME) based on the MiSeq platform |
| Gabriela Batitucci2023 | NR | NR | NR | NR | NR | —— |

**8. supplementary materials Table S7. Summary of Dietary and Medication Control**

| Study | Control Type | Details |
| --- | --- | --- |
| Beals. 2023 | Diet | Individualized dietary and behavioral education was provided during the intervention. All meals were prepackaged and plant-based, low in fat, sodium, and refined carbohydrates, and high in complex carbohydrates from vegetables, fruits, whole grains, seeds, and legumes. The energy distribution was approximately 70% carbohydrates, 15% fat, and 15% protein. |
|  | Medication | Participants were excluded if they were taking medications that could affect outcomes, consuming excessive alcohol (>21 units/week for men, >14 units/week for women), pregnant, or breastfeeding. |
| Ky Young Cho. 2021 | Diet | A lifestyle and dietary habits questionnaire was administered, with no significant differences between groups. Participants received 1–2 personalized “must-follow” recommendations (e.g., eat breakfast, avoid sugary drinks, reduce processed animal-fat foods, or prolong meal duration). |
|  | Medication | Individuals with congenital heart disease, chronic inflammatory bowel disease, chronic liver or kidney disease were excluded. Participants must not have used antibiotics, probiotics, or corticosteroids within 1 month prior to the intervention. |
| Owen Cronin2018 | Diet | All participants were instructed to maintain their usual ad libitum diet during the intervention and to refrain from taking additional vitamin, dietary, or herbal supplements |
|  | Medication | Exclusion criteria included a history of substance abuse, coronary artery disease, congenital heart disease, or any other cardiovascular conditions. Participants requiring antibiotics during the intervention were withdrawn from the study. |
| Jesús F García-Gavilán2024 | Diet | Dietary assessment was conducted by a dietitian at baseline and annually using a validated 143-item food frequency questionnaire (FFQ). |
|  | Medication | Sociodemographic data, medical history, and leisure-time physical activity (assessed via the Regicor questionnaire) were recorded to control for non-dietary confounders |
| Jukka E. Hintikka2023 | Diet | Diet was assessed via questionnaire, and participants were advised to maintain their habitual free-living diet. Energy and macronutrient intake were analyzed using Micro-Nutrica software based on 3-day food records (2 weekdays and 1 weekend), including meal timing, food and beverage types, and quantities. |
|  | Medication | antibiotic use within the past 2 months; major inflammatory gastrointestinal disorders; significant eating disorders; diagnosed type 1 or type 2 diabetes; cardiovascular diseases other than hypertension; hypothyroidism or other endocrine disorders that may affect training or outcomes; musculoskeletal conditions impairing training or testing capacity. |
| Libuša Nechalová2024 | Diet | All participants received detailed guidance and counseling on lifestyle adherence to adjust caloric intake. |
|  | Medication | Exclusion criteria included antibiotic/probiotic use for two consecutive weeks or within the past two months, and recent acute illness (e.g., infection, fever, upper respiratory tract infection, chronic inflammation, or autoimmune disease). |
| L.Torquati2022 | Diet | Participants were instructed to maintain their habitual diet throughout the intervention period. |
|  | Medication | Participants were required to have stable medication use and body weight (<5 kg variation) in the 3 months prior to enrollment. Individuals with any condition listed in the ACSM guidelines were excluded from the main trial. Those who had taken antibiotics or experienced gastrointestinal inflammation within the past 3 months were excluded from the sub-study. |
| ALLEN.JACOB M.2018 | Diet | Participants maintained habitual dietary patterns, including alcohol, caffeine, and supplement intake; 3-day menus were analyzed using the NDSR system. |
|  | Medication | Inclusion criteria required the absence of metabolic or gastrointestinal disorders, no pregnancy or lactation, no use of medications affecting gut function, and no antibiotic use for at least 3 months prior to and during the study. |
| Martin S. Lietzén2024 | Diet | Participants were required to complete dietary diaries during the intervention. |
|  | Medication | Exclusion criteria included mental disorders, poor compliance, eating disorders, excessive alcohol use, active peptic ulcers, pregnancy, MRI contraindications, or other conditions potentially affecting participant safety or outcome interpretation. |
| Runtan Cheng2022 | Diet | A standardized diet was provided during intervention (30–40% of total daily energy), consisting of 37–40% carbohydrates (including 9–13g fiber), 35–37% fat (10% SAFA, 15–20% MUFA, ≤10% PUFA), 25–27% protein, plus 5g soluble fiber supplement. |
|  | Medication | Screening questionnaire ensured eligibility: alcohol consumption <21 drinks/week (men), <14 drinks/week (women); no chronic cardiovascular, severe musculoskeletal, or gastrointestinal disease; no extreme dietary patterns. |
| John M. A. Cullen2024 | Diet | Participants were instructed to maintain habitual diet and lifestyle throughout the intervention to avoid confounding effects. |
|  | Medication | Medical history questionnaire was used to screen diseases and medications; participants with antibiotic use in the past 6 months or treatment for arterial disease, heart failure, or dyslipidemia were excluded. |
| Martha Guevara‐Cruz2019 | Diet | Dietary intake was recorded during the intervention and monitored via telephone supervision. |
|  | Medication | Strict exclusion of recent medication and GI disorders: use of pro-motility agents within 4 weeks, antibiotics or symbiotic-related therapy within 2 months, high-fiber intake (>15 g/day); participants with GI dysfunction, IBD, IBS, or history of major abdominal surgery were excluded. |
| Timo Kern2020 | Diet | Dietary intake was recorded for 3 weekdays and 1 weekend day with weighed food records to analyze energy and macronutrient intake; no other lifestyle changes were required during the intervention. |
|  | Medication | Individuals taking prescription medications (except contraceptives) or with a first-degree family history of type 2 diabetes were excluded. |
| Zong yu Lin 2023 | Diet | The intervention was delivered via a self-developed app with dietary and physical activity reminder functions; participants received daily prompts to log food intake and exercise. |
|  | Medication | Exclusion criteria included participation in other weight loss studies, secondary obesity (due to medication or disease), hypertension, diabetes or other cardiovascular diseases, and contraindications to exercise. |
| Yao Wang2024 | Diet | Participants were instructed to maintain their habitual dietary habits during the intervention. |
|  | Medication | Exclusion criteria included neurological, musculoskeletal, or cardiorespiratory diseases; engaging in >2 sessions/week of vigorous exercise or dieting within 3 months prior to recruitment; and any medication use, especially antibiotics, within 1 week before the study. |
| Shao dong Wei 2021 | Diet | Individualized dietary plans were provided (45–60% carbohydrates, 15–20% protein, 20–35% fat, <7% saturated fat); energy intake was restricted during the first 4 months, with dietary counseling delivered by clinical dietitians. |
|  | Medication | To minimize bias, endocrinologists standardized antidiabetic, lipid-lowering, and antihypertensive treatments based on predefined therapeutic targets and algorithms. |
| Fei Zhong2022 | Diet | Participants were instructed to maintain their habitual dietary patterns during the intervention. |
|  | Medication | Exclusion criteria included prior treatment for malignancy, history of acute myocardial infarction, heart failure, or stroke; current use of antihypertensive or lipid-lowering medications; any condition limiting physical activity or safe exercise capacity; and antibiotic use within one month prior to the study. |
| Gabriela Batitucci2023 | Diet | Participants were instructed to keep dietary records and report them to the study investigators. |
|  | Medication | Detailed exclusion criteria were established, including individuals with diabetes, hypertension, dyslipidemia, heart disease, osteoarthritis, or gastritis, as well as those who smoked, abused alcohol, or were on regular medication. |
| DUPUIT, MARINE2022 | Diet | At baseline and week 12, participants completed a 5-day dietary record (3 weekdays and 2 weekend days), assessed by dietitians using Nutrilog® software (Marans, France); telephone support was provided if needed. |
|  | Medication | Dietary habits and physical activity were stable for at least 3 months prior to the intervention. Exclusion criteria included contraindications to vigorous exercise, joint pain, hormone replacement therapy, and antibiotic use within the past 3 months. |
